# Associations of 5-year changes in alcoholic beverage intake with 5-year changes in waist circumference and BMI in the Coronary Artery Risk Development in Young Adults (CARDIA) study

**DOI:** 10.1371/journal.pone.0281722

**Published:** 2023-03-08

**Authors:** J. Lauren Butler, Penny Gordon-Larsen, Lyn M. Steffen, James M. Shikany, David R. Jacobs, Barry M. Popkin, Jennifer M. Poti

**Affiliations:** 1 Department of Nutrition, Gillings School of Global Public Health, University of North Carolina at Chapel Hill, Chapel Hill, North Carolina, United States of America; 2 Nutrition and Foods Program, School of Family and Consumer Sciences, Texas State University, San Marcos, Texas, United States of America; 3 Division of Epidemiology and Community Health, School of Public Health, University of Minnesota, Minneapolis, Minnesota, United States of America; 4 Division of Preventive Medicine, University of Alabama at Birmingham, Birmingham, Alabama, United States of America; National Dental Research Institute Singapore / Duke NUS Medical School Singapore, SINGAPORE

## Abstract

**Objective:**

This study aimed to shed light on contradictory associations of alcohol intake with waist circumference (WC) and body mass index (BMI) by examining 5-yr changes in alcohol intake in relation to 5-yr WC and BMI changes.

**Methods:**

This prospective study included 4,355 participants (1,974 men and 2,381 women) enrolled in the Coronary Artery Risk Development in Young Adults (CARDIA) study at baseline (1985–1986) and followed over 25 years (2010–2011). Longitudinal random effects linear regression models were used to test whether changes in drinking (defined categorically) as starting to drink, increasing, decreasing, stable drinking or stopping drinking (versus stable non-drinking) over a series of 5-yr periods were associated with corresponding 5-yr WC and BMI changes. Associations with 5-yr changes (defined categorically as starting, stable or stopping) in drinking level (i.e., light/moderate and excessive) and 5-yr changes (defined categorically as increasing, no change, or decreasing) by beverage type (i.e., beer, wine and liquor/mixed drinks) were also examined.

**Results:**

In men, compared to stable non-drinking, decreasing total alcohol intake was associated with lower 5-yr WC (β:-0.62 cm; 95% CI: -1.09, -0.14 cm) and BMI gains (β:-0.20 kg/m^2^; 95% CI: -0.30, -0.03 kg/m^2^) and stopping excessive drinking was associated with lower 5-yr WC gains (β:-0.77 cm; 95% CI: -1.51, -0.03 cm). In women, compared to those with stable non-drinking habits, starting light/moderate drinking was associated with lower 5-yr WC (β: -0.78 cm; 95% CI: -1.29, -0.26 cm) and BMI gains (β:-0.42 kg/m^2^; 95% CI: -0.64, -0.20 kg/m^2^). Increasing wine intake was associated with a lower 5-yr BMI gain (β:-0.27 kg/m^2^; 95% CI: -0.51, -0.03 kg/m^2^). Decreasing liquor/mixed drink (β:-0.33 kg/m^2^; 95% CI: -0.56, -0.09 kg/m^2^) intake was associated with lower 5-yr WC (β:-0.88 cm; 95% CI: -1.43, -0.34 cm) and BMI (β:-0.33 kg/m^2^; 95% CI: -0.56, -0.09 kg/m^2^) gains.

**Conclusions:**

Associations of alcohol intake with obesity measures are complex. In women, wine and liquor/mixed drink intakes had contrasting associations with WC and BMI change. In men, decreasing weekly alcoholic beverage intake with an emphasis on stopping excessive consumption may be beneficial in managing WC and BMI gains.

## Introduction

An increasing trend in energy consumed from alcoholic beverages coupled with secular increases in waist circumference (WC) and body mass index (BMI) have been reported in the US over the past two decades [1–3]. The *Dietary Guidelines for Americans*, *2020–2025* (DGA) explicitly state that “alcoholic beverages are not a component of the United States Department of Agriculture (USDA) Dietary Patterns and that regular consumption of alcoholic beverages can make it challenging for adults to meet food group and nutrient needs while not consuming excess calories” [4]. However, the DGA do not mention associations of alcohol intake with WC or BMI. In fact, results of studies of alcohol intake with measures of weight status are inconsistent. Positive, null and negative associations of alcohol intake with WC, BMI, and changes in WC and BMI have been reported. Residual confounding, selection bias and variation in associations by drinking level and alcoholic beverage type have been cited as potential contributors to contradictory findings [5–8].

Residual confounding by unmeasured characteristics that differ within and across drinking categories may underlie inconsistent findings [5,9,10]. In the US, people who don’t drink have been reported to engage in less physical activity, consume more energy and belong to lower socioeconomic subgroups as compared to those that drink [8,11]. In one study of Northern Californians, those who reported heavy drinking included higher proportions of persons who preferred drinking beer and liquor as compared to the proportion of those who preferred wine [12]. In a 2021, Mendelian randomization analysis of 334,507 white British adults Zhou et. al. found that socioeconomic status significantly mediated the relationship between educational attainment and alcoholic beverage choice. Those with higher educational attainment may be more likely to be exposed to messages that red wine is associated with long-term health benefits. Leading to higher consumption of wine as compared to their lower socioeconomic status counterparts [13,14]. Among US adults, wine drinking has been associated with higher educational attainment and higher intakes of food and beverage groups supported by the DGA [4,8,12,15]. In a systematic review of associations of alcoholic beverage preference with dietary habits, Sluik et. al concluded that those in Western and Mediterranean countries who prefer to drink beer and liquor generally have less healthy dietary habits [15]. For example, in one US-based study, beer and liquor drinkers had higher adjusted mean total fat and lower adjusted mean dietary fiber intakes as compared to those who preferred to drink wine [16]. Despite associations with alcohol intake and measures of obesity, dietary intake and physical activity have been cited as key omitted confounders in epidemiologic studies of alcohol and obesity outcomes [5,17]. People may self-select into alcohol consumption behavior patterns based on socio-demographic characteristics and inherent individual traits [18,19]. If unaccounted for, residual confounding and self-selection may bias associations of alcohol intake with WC and BMI and contribute to inconsistencies in the alcohol and obesity literature [8,9,20].

In addition, variation in associations by drinking level and alcoholic beverage type may add to the mixed literature. Positive and null associations of excessive drinking and BMI gains have been reported [5,21,22]. There is evidence that stable heavy drinking, in men, and maintaining stable light or moderate drinking, in women, may underlie positive and negative associations of within-person changes in total alcohol intake with WC and BMI change in men and women, respectively [23,24]. With regard to alcoholic beverage type, a standard drink contains roughly 14 grams of alcohol. Yet, energy content, percent alcohol by volume (%ABV) and bioactive components vary considerably across beverage types. Unlike liquor, beer and wine contain bioactive compounds (e.g., polyphenols and resveratrol), tend to have a lower %ABV, and can have fewer calories than liquor served with a mixer [25–27]. Positive and negative associations of beer intake and changes in beer intake with weight and BMI gains have been found [17,28]. While wine intake has been negatively associated with weight gain, positive associations with liquor consumption have been reported for both sexes [25,29]. The results are limited and inconclusive regarding associations of within-person changes in alcohol consumption levels and alcoholic beverages by type (e.g., decreasing beer or wine intake) and changes in WC and BMI [5,17,24,25,29–37]. Furthermore, to our knowledge no study has examined within-person changes in drinking level in relation to changes in WC in men and women [5,8].

To address these gaps in the literature, we used time-varying data on alcoholic beverage intake, diet, physical activity and socio-demographic covariates over 25 years from the Coronary Artery Risk Development in Young Adults (CARDIA) study to determine whether changes in WC and BMI differ between people who drink and those that do not. Using within-person change analyses to control for time-invariant unobserved individual characteristics, we examined changes in drinking level and changes in intake by beverage type in relation to changes in WC and BMI. We hypothesized that starting to consume alcohol in excess over a 5-yr period within the 25-year study period would be positively associated with WC and BMI change. Additionally, we hypothesized that 5-yr increases in liquor/mixed drinks would be positively associated with 5-yr WC and BMI change [8]. Findings from our study could provide evidence to support clinicians in discussing the DGA guidance for alcohol intake as part of weight-related health care recommendations [4].

## Materials and methods

CARDIA is an ongoing, prospective study of the determinants and evolution of cardiometabolic risk starting in young adulthood. Participants for the baseline examination were randomly selected and recruited by telephone or door to door from census tracts in the four field centers: Minneapolis, MN and Chicago, IL, by telephone exchanges within the Birmingham, AL city limits, and from lists of the Kaiser-Permanente Health Plan membership in Berkeley, CA. Although the source populations and methods of recruitment varied slightly, all centers adhered to the following basic eligibility criteria: 18–30 years of age, Black or white race, and permanent residential address in the target areas. Individuals living with a long-term illness or disability that would prevent participation and pregnant women and those who were up to 3 months postpartum were excluded from recruitment [38,39]. A total of 5,115 young adults aged 18–30 years were enrolled at baseline in 1985–1986 (Exam Year 0) with balance according to race (Black and white), sex, education (≤high school and >high school), and age (18–24 and 25–30 years) from the population in each of the four metropolitan areas. Follow-up examinations occurred in 1987–1988 (Exam Year 2), 1990–1991 (Exam Year 5), 1992–1993 (Exam Year 7), 1995–1996 (Exam Year 10), 2000–2001 (Exam Year 15), and 2005–2006 (Exam Year 20) and 2010–2011 (Exam Year 25); retention at each exam year was 91%, 90%, 86%, 81%, 79%, 74%, 72% and 72%, respectively. The CARDIA study methods are described in detail elsewhere [38,39]. Each study participant provided written informed consent, and data were collected under protocols approved by the Institutional Review Boards at each study center and at the University of North Carolina at Chapel Hill [8]. The current study included CARDIA exam years 0, 5, 10, 15, 20 and 25. We excluded participants with only one of the six exams used in this analysis. As has been done in previous studies, to minimize bias resulting from illness that may affect body weight, we excluded participants with hypertension, diabetes or cancer at exam year 0 [32,40]. We further restricted the analytic sample to those with data on diabetes, hypertension, or self-reported cancer diagnoses at each exam and those with waist circumference and BMI data at exam year 0. For individuals included in the primary analytic sample, observations were excluded at given exam years if participants were pregnant or breastfeeding or had implausible energy intakes (<600 kcal/d or >6000/d kcal for women and <800 kcal/d or >8000 kcal/d for men) at any exam or if they were missing exposure, outcome, or covariate data at a given exam year. We censored observations for participants with diabetes, hypertension, or self-reported cancer during follow-up at the year in which the disease was reported. To facilitate sensitivity analyses, a secondary analytic dataset was created in accordance with the primary analytic dataset exclusion criteria except for censoring on diabetes, hypertension, or self-reported cancer. The secondary dataset is described in the (S1 File) [8,32,40].

### Measures

Participant and interviewer-administered questionnaires were used to obtain sociodemographic, lifestyle, medical and behavioral data. Education, income, and marital status were assessed using the CARDIA Sociodemographic Questionnaire. At each exam year, participants were queried on the highest degree earned (“< HS”; “HS or equivalency”; “Associate Degree”; “Bachelor’s Degree”; “Master’s Degree”, “Doctoral Degree, Professional Degree (e.g. medical doctor, Doctor of Dental Surgery))” and the years of education completed (0 to 20+ years). When degree data was missing, the years of education variable was used to categorize participants: “< HS” (<12 years), “HS or equivalency” (12 years), “Associate, Bachelor’s, Graduate or Professional Degree” (>12 years). Income data was not collected at exam year 0 or 2. From exam year 5 to 10 participants were asked to report annual family income categorically as “< $5,000”; “$5,000 to $11,999”; “$12,000 to $15,999”; “$16,000 to $24,999”; “$25,000 to $34,999”; “$35,000 to $49,999”; “$50,000 to $74,999”; “≥ $75,000”. In exam year 15, the “≥ $75,000” category was disaggregated into “$75,000 to $99,999” and “≥$100,000”. In this study, the income data from exam year 5 was carried backward; the first and second value of income were therefore the same while the third, fourth, fifth and sixth values changed based on participants’ reports over time. At all exam years, participants were asked to identify as “Married”; “Divorced”; “Separated”; “Never Married” or “Other”.

Smoking status was determined from the CARDIA Tobacco Use Questionnaire. Respondents were asked: (1) “Have you ever used any tobacco product such as cigarettes, cigars, tobacco pipe, chewing tobacco, snuff or nicotine chewing gum?”; (2) “Have you ever smoked cigarettes regularly for at least three months? By “regularly” we mean at least 5 cigarettes per week almost every week”. Those that reported “no” to regular tobacco use were categorized as “people who never smoked”. Respondents who answered “yes” to smoking regularly (smoking at least five cigarettes per week, almost every week for at least 3 months) completed a follow-up tobacco use questionnaire and were asked “Do you still smoke cigarettes regularly?”. Those that answered “no” were coded as “people who formerly smoked" and those that answered “yes” were coded as “people who currently smoke”.

Physical activity was assessed using the CARDIA Physical Activity Questionnaire (PAQ), a validated and reliable assessment of physical activity [41]. In brief, the PAQ queries participants on performance of 13 different physical activities, the total number of months of performance per year and how many months the activity has been performed for at least a specific number of hours per week during the month. An intensity level is assigned to each activity according to the number of kilocalories expended in one minute of activity and patterned using methods of coding intensity based on the formulation developed by Reiff et al [42]. Summary scores are calculated based on energy expenditure in heavy intensity and moderate intensity activities. Because no interpretation as caloric expenditure per week is available the summary score is left unaltered in “Exercise Units” (EU) per week [41].

Dietary intake data for this study was derived from a validated interviewer-administered comprehensive diet history questionnaire administered at exam years 0, 7, and 20 [43,44]. Interviewers asked participants open-ended questions about dietary consumption during the past month within 100 food categories, referencing 1609 separate food items in years 0 and 7 and several thousand food items in year 20. Follow-up questions addressed serving size, frequency of consumption and common additions to foods. Diet history data used codes of the University of Minnesota Nutrition Coordinating Center (NCC) and foods were placed into 166 food groups using the food grouping system developed by the NCC. Then, trained personnel translated the pre-coded dietary items to estimate the individual nutrient intake using the Nutrition Data System for Research (NDSR, versions 10, 20, and 36 for year 0, year 7, and year 20, respectively) [45,46]. A study evaluating the reliability and comparative validity of the CARDIA diet history was conducted using two dietary histories to assess reliability and seven telephone-assessed 24-hour dietary recalls to assess comparative validity. Results of this study indicate that the CARDIA diet history is a reliable and valid dietary survey method for obtaining estimates of usual dietary intake [43,47]. For those years that dietary intake was not assessed, data from the previous year was carried forward. In the current study, to characterize total dietary composition, the percent contribution of each macronutrient (% carbohydrate, % protein and % fat) was calculated based on the percent contribution of each macronutrient to total energy intake. We also report total non-alcoholic energy, calculated by excluding the energy from alcoholic beverages. Diet quality was defined using the a-priori diet quality score previously developed and used as a valid predictor of clinical cardiovascular disease, myocardial infarction and diabetes [48–50]. This summary score of diet quality was constructed by classifying 46 food groups according to investigator ratings of hypothesized health effects. Twenty food groups were identified as beneficial, 13 as adverse, and 13 as neutral. Within the CARDIA dataset, this ‘a-priori’ diet quality score has been associated with lipid peroxidation and age, gender, race and education [51,52]. In this study, alcoholic beverages were excluded from the calculation of the diet quality score.

Pregnancy and breastfeeding status, self-reported cancer and hypertension data were obtained from the CARDIA Medical History Questionnaire at each exam year. Women who answered “yes” to the questions “Are you pregnant?” or “Are you breastfeeding?” were coded as pregnant or breastfeeding. Respondents were asked “Has a doctor or nurse ever told you that you have cancer?”. Those that answered “yes” were coded as having a history of self-reported cancer. Hypertension was defined as a ‘‘yes” response to the question ‘‘Has a doctor ever told you that you have hypertension?” or average measured systolic or diastolic blood pressure (SBP/DBP) exceeding ≥ 140/≥90 mm Hg. Resting seated blood pressure was measured 3 times and the average of the 2nd and 3rd readings was used to define hypertension. Diabetes was determined based on a combination of measured fasting glucose levels (≥7.0 mmol/L and ≥126 mg/dL) at exam years 0, 7, 10, 15, 20, or 25; self-report of oral hypoglycemic medications or insulin at exam years 0, 7, 10, 15, 20, or 25; a 2-h postload glucose ≥11.1 mmol/L (≥200 mg/dL) during a 75-g oral glucose tolerance test at exam years 10, 20, and 25; or an HbA1c ≥6.5% at exam years 20 and 25. Blood was drawn by venipuncture and processed at the central laboratory according to a standard protocol. Glucose was assayed at baseline using the hexokinase ultraviolet method by American Bio Science Laboratories (Van Nuys, CA) and at years 7, 10, 15, 20, and 25 using hexokinase coupled to glucose-6-phosphate dehydrogenase (Linco Research, St. Louis, MO). Glucose values at follow-up were recalibrated to year 0 glucose values. HbA1c was measured using the Tosoh G7 high-performance liquid chromatography method at years 20 and 25. For clinical measures, participants were asked to fast for at least 12 hours before each examination and to avoid smoking or engaging in heavy physical activity for at least 2 hours.

All data were collected by trained staff with a standardized protocol. All exam materials can be found on the public CARDIA website: https://www.cardia.dopm.uab.edu/. The analytic code for creating the smoking status, physical activity score, hypertension and diabetes variables used in this study is publicly available on the CARDIA website: https://www.cardia.dopm.uab.edu/study-information/derived-variables-from-cardia-data.

### Alcoholic beverage consumption

Alcoholic beverage consumption was assessed at each exam using the CARDIA Alcohol Use Questionnaire (AUQ) that queried participants on annual, monthly, weekly and daily alcoholic beverage intake. Alcoholic beverage consumption was defined based on the following questions: “Did you drink any alcoholic beverages in the past year?”; “How many drinks of wine (5 oz glass) do you usually have per week?”; “How many drinks of beer (12 oz glass) do you usually have per week?”; “How many drinks per week do you usually have of hard liquor (1 1/2 oz)?” To our knowledge there is no validated questionnaire available to measure usual alcoholic beverage intake in epidemiologic studies. However, in exploratory analyses, we found that alcoholic beverage intake (milliliters of alcohol per day) estimated from the AUQ was strongly correlated with mean alcohol intake (grams per day) estimated from the validated CARDIA diet history at exam years 0 (r = 0.77; p < .001); year 7 (r = 0.71; p < .001); and year 20 (r = 0.79; p < .001).

To describe the distribution of socio-demographic and lifestyle characteristics of people who drank compared to those who didn’t drink at baseline, participants were categorized into sex-specific drinking categories using alcoholic beverage intake at exam year 0. Category definitions were based on the National Institute on Alcohol Abuse and Alcoholism (NIAAA) guidance on drinking levels [34,53–56]. Based on the sum of the usual intake of beer, wine, and liquor/mixed drinks per week (drinks/wk) as reported on the AUQ at exam year 0, men were classified as “non-drinker”, “light drinker” (<7 drinks/wk), “moderate drinker” (7 to 14 drinks/wk), or “excessive drinker” (>14 drinks/wk), and women were classified as “non-drinker”, “light drinker” (<4 drinks/wk), “moderate drinker” (4 to 7 drinks/wk), or “excessive drinker (> 7 drinks/wk) [8,53].

#### Changes in total alcohol intake

Alcoholic beverage intake data were collected at all examinations. To capture 5-yr changes in alcohol intake, we chose to use alcohol intake data from the six examinations administered with 5-yr time intervals from one exam to the next (i.e., exam years 0, 5, 10, 15, 20 and 25). Participants were categorized by the 5-yr change in total drinks/wk from one exam year to the next, relative to drinking status at the previous exam, as follows: “Stable non-drinking” (0 drinks/wk at previous and current exam), “Start drinking” (change from 0 drinks/wk at previous exam to > 0 drinks/wk at current exam), “Increase drinking” (drinks/wk at previous exam < drinks/wk at current exam), “Stable drinking” (drinks/wk > 0 and drinks/wk at previous exam equal to drinks/wk at current exam), “Stop drinking” (change from >0 drinks/wk at previous exam to 0 drinks/wk at current exam), “Decrease drinking” (drinks/wk at previous exam > drinks/wk at current exam) [8].

#### Changes in drinking level

To investigate associations of 5-yr changes in drinking level with 5-yr changes in WC and BMI in the 25 year study period, participants were categorized by the change in NIAAA-based drinking levels from one exam year to the next, relative to drinking status at the previous exam, as follows: “Stable non-drinking” (0 drinks/wk at previous and current exam), “Start light/moderate drinking” (non-drinker at previous exam and light (0> to < 4 drinks/wk in women; 0> to <7 drinks/wk in men) or moderate (4 to 7 drinks/wk in women; 7 to 14 drinks/wk in men) drinker at current exam), “Start excessive drinking” (non-, light or moderate drinker at previous exam and excessive (> 7 drinks/wk in women; >14 drinks/wk in men) drinker at the current exam), “Stable light/moderate drinking” (light or moderate drinker at previous exam and light or moderate drinker at current exam), “Stable excessive drinking” (excessive drinker at previous exam and excessive drinker at current exam), “Stop light/moderate drinking” (light or moderate drinker at previous exam and non-drinker at current exam), “Stop excessive drinking” (excessive drinker at previous exam and non-, light or moderate drinker at the current exam) [8].

#### Changes in alcoholic beverage type

To examine associations of 5-yr changes in beer, wine and liquor/mixed drink intake with 5-yr changes in WC and BMI over the 25-year period, participants were categorized relative to drinking status at the previous exam year and according to weekly consumption of each beverage type as follows: “Stable non-drinking” (0 drinks/wk at previous and current exam), “Increase” (beer, wine or liquor/mixed drinks/wk at previous exam < beer, wine or liquor/mixed drinks/wk at current exam), “No change” (no change in beer, wine or liquor/mixed drinks/wk intake from previous to current exam), “Decrease” (beer, wine or liquor/mixed drinks/wk at previous exam > beer, wine or liquor/mixed drinks/wk at current exam) [8].

### Anthropometrics

At each exam, height, weight, and WC were measured in replicate in light clothing without shoes according to standardized protocol [2,57]. Height was measured to the nearest 0.2 cm via portable stadiometer (Seca Corporation, Chino, CA), and weight was measured to the nearest 0.1 kg via calibrated balance beam scale. WC was measured midway between the iliac crest and the lowest lateral portion of the rib cage (anteriorly at the point midway between the xiphoid process of the sternum and the umbilicus) using a Seca tape measure, and an average of 2 measures to the nearest 0.5 cm was used. BMI was calculated as weight in kilograms divided by height in meters squared (kg/m^2^) [8].

### Statistical analyses

All data analyses were conducted using Stata, version 14 (StataCorp, College Station, TX).

Initial unadjusted descriptive analyses tested whether demographic, socioeconomic, and behavioral characteristics and anthropometric outcomes at baseline in 1985–1986 among people who did not drink differed from those who did in each NIAAA-based drinking category. Chi square tests were used to determine differences in the distribution of categorical covariates and analysis of variance (ANOVA) was used to test means of continuous covariates.

Longitudinal random effects linear regression models with an exchangeable correlation structure, which account for correlation between repeated measures within individuals across time with a random intercept for each individual, were used to determine whether 5-yr changes in alcohol intake were associated with changes in WC and BMI during the same time period [8]. All models adjusted for several demographic, socioeconomic, and behavioral factors that were assessed at each examination. These confounders were chosen based on their well-established associations with alcohol intake, WC and BMI and to be comparable to other similar studies of alcohol intake and obesity measures [17,25,58,59]. The time invariant covariates were: baseline age (18–24 years or 25–30 years); baseline WC (when change in WC was the outcome) or baseline BMI (when change in BMI was the outcome); and race (Black or White). We adjusted for time-varying education (< = high school; (HS) diploma; >HS); income (≤$24,999; $25,000–$74,999; ≥$75,000); and smoking status (people who never smoked, people who formerly smoked or people who currently smoke) at the start of each 5-yr interval. Because < 10% of participants within each drink change category experienced changes in income, education or smoking status, we did not adjust for changes in these covariates within each 5-yr period. Modeling income, education and smoking status as time-varying addressed confounding from variation in these covariates that occurred over the 25-year study period. We adjusted for time-varying changes in marital status (stable single, stable married or change in marital status); physical activity score (continuous); and diet quality score (continuous) within each 5-yr period.

An example of the linear models that regressed 5-yr WC or BMI change on 5-yr change in total alcoholic drinks/wk over the same time period (categorized with stable non-drinkers as the referent group) is shown below.

Variables that are calculated as a change over the 5-yr period are preceded by δ.


$${\delta Y}_{i\left(t,t+5\right)}=a+{b\delta D}_{i\left(t,t+5\right)}+{cX}_{i}+{dC}_{it}+{e\delta Z}_{i(t,t+5)}+\varepsilon_{it}+\mu_{i}$$

For this model, δY_i(t,t+5)_ is the 5-yr change in WC or BMI of person i during time interval t to t + 5;

a is the population-level intercept;

δD_i(t,t+5)_ is the 5-yr change in alcoholic beverage intake of person i during time interval t to t + 5, defined by categories start, increase, stable, stop or decrease;

X_i_ represents time invariant covariates for person i (including baseline WC or BMI measure depending on the outcome modeled);

C_it_ represents one set of time varying covariates, specifically education, income and smoking status, for person i at time t, the beginning of the interval;

δZ_i(t,t+5)_ represents changes in a separate set of time varying covariates, including marital status, physical activity score and diet quality score, of person i during time interval t to t + 5;

ε_it_ is the usual random disturbance, estimating the within person variability in 5-year change in WC or BMI; and

μ_i_ is an individual-specific disturbance term, estimating the between person variability in 5-year change in WC or BMI.

Separate models were used to test whether associations between 5-yr changes in NIAA-based drinking level and 5-yr changes in WC and BMI differed from that of stable non-drinkers.

Separate models were also used to test whether 5-yr changes in each type of alcoholic beverage in drinkers were associated with 5-yr changes in WC and BMI. Models for each beverage type were adjusted for time-varying continuous changes in intake of each other alcoholic beverage within each 5-yr interval.

Because the existing literature suggests that alcohol intake has differential associations with adiposity among men and women, all analyses were stratified by sex [8]. All results (in men and women) are compared with the 5-yr WC and BMI gains observed among those with stable non-drinking patterns. Results were considered significant at p<0.05.

## Results

**Fig 1:**
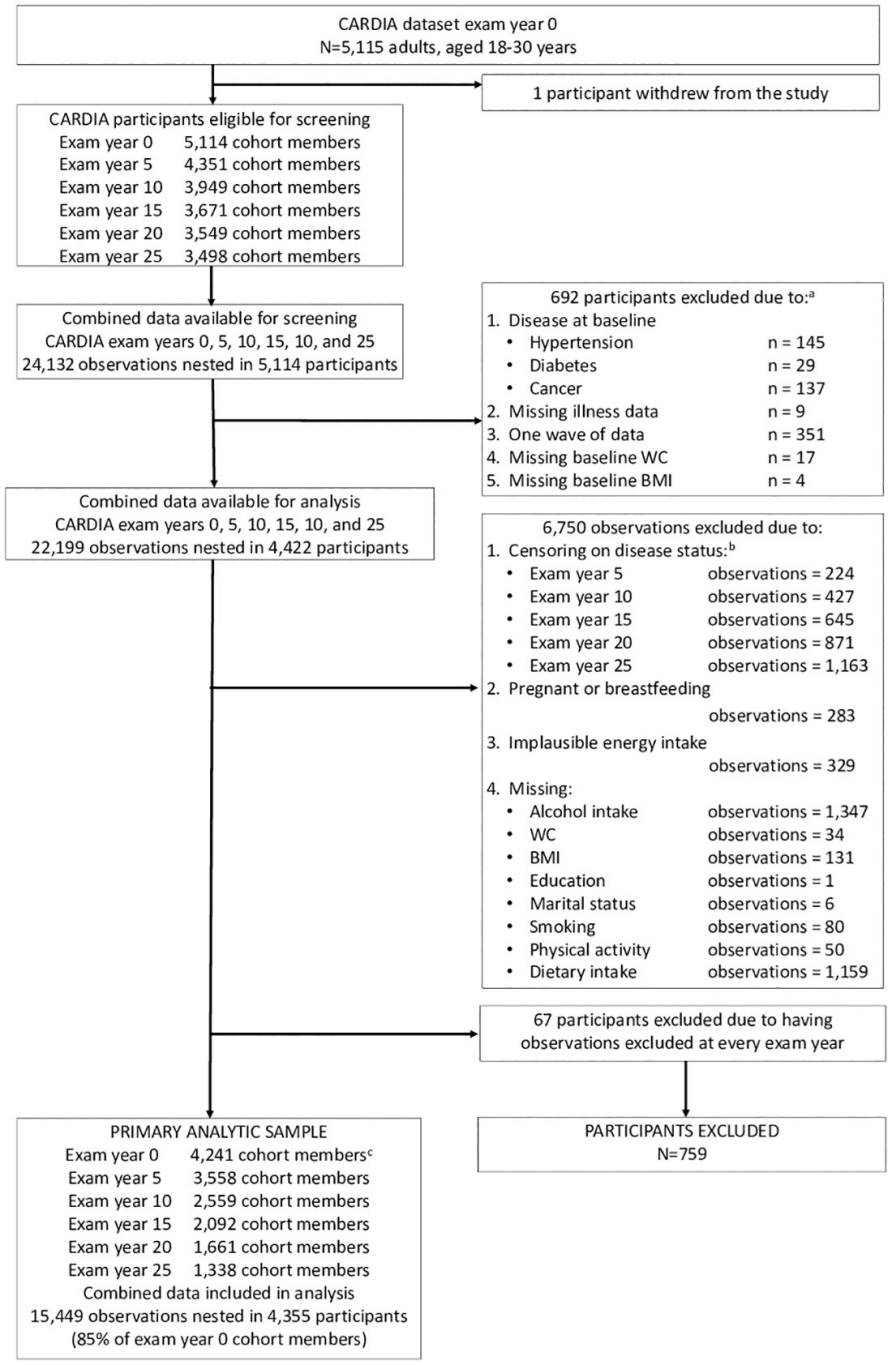
STROBE flow diagram of study participant inclusion and exclusion criteria. ^a^ Participants (n). ^b^ Observations censored for participants with diabetes, hypertension or self-reported cancer during follow-up at the year in which the disease was reported. ^c^ 114 participants included in the final analytic sample had excluded observations at exam year 0.

For this study 5,114 participants (observations = 24,132) were screened for eligibility. 692 participants were excluded due to disease diagnoses or missing WC or BMI data at baseline; missing disease data at any exam year or having only one wave of data. After excluding participants who did not meet initial study inclusion criteria 4,422 participants (observations = 22,199) remained. 6,750 observations were excluded due to censoring, pregnancy or breastfeeding, implausible energy intakes or missing exposure, outcome, or covariate data. 67 participants were excluded due to excluded observations at every exam year. In total 759 participants were excluded, and our primary analytic sample included 4,355 participants (observations = 15,499) (Fig 1). The secondary analytic dataset, that did not include censoring on diabetes, hypertension or self-reported cancer included n = 4,377 participants (observations = 18,671) (S1 File).

Excluded participants were more likely to be Black people, living with obesity, currently smoking and belong to the lowest education subgroup at exam year 0 as compared to those included in the primary analyses (S1 Table).

### Baseline characteristics

Men who do not drink had higher proportions of Black people and people who do not smoke than moderate and excessive drinking men (Table 1) and women (Table 2). The proportion of adults aged 25 to 30 years was lower among those who do not drink as compared to all drinking categories. 13.8% of men and 9.9% of women reported drinking excessively. In men, the proportion of those in the lowest education group was higher among people who don’t drink and those that drink excessively than people with light and moderate drinking frequencies. Men who drink excessively had significantly higher WC compared to people who do not drink. In women, the proportion of those who were married was higher among those who did not drink and drink only lightly as compared to those who drink excessively. Women who did not drink had significantly higher WC than those who did drink. People who didn’t drink had significantly lower total energy intake coupled with lower diet quality and physical activity as compared to all drinking levels among men and women.

**Table 1:** Baseline characteristics of men in the CARDIA study 1985–1986, according to baseline alcoholic beverage intake (total drinks/wk)^a^.

	Total drinks/wk				
Baseline Characteristics	Non-drinker	0> to <7	7 to 14	>14	*p*
**N**	558	700	408	267	
**(%)**	28.9	36.2	21.1	13.8	
**Race (%)**					
White	43.7	53.7	59.3	59.9	<0.001
Black	56.3	46.3	40.7	40.1	
**Age cohort (%)**					
18–24 yrs	50.4	47	36.5	39	<0.001
25–30 yrs	49.6	53	63.5	61	
**Marital status (%)**					
Single, Widowed or Divorced	75.4	78.3	82.8	84.3	0.006
Married or Cohabitating	24.6	21.7	17.2	15.7	
**Smoking status (%)**					
Never smoked	71.7	59.1	47.1	34.1	<0.001
Formerly smoked	10.6	11.1	17.4	16.1	
Currently smoke	17.7	29.7	35.5	49.8	
**Physical Activity (EU)**^b^ **(mean ± SE)**	469.8 ± 299.2	513.5 ± 304.6	542.1 ± 311.5	547 ± 322.5	0.001
**Education (%)**					
≤ High School	72.0	64.9	62	72.3	0.001
> High School	28.0	35.1	38.0	27.7	
**Income**^c^ **(%)**					
≤ $24,999	38.7	36.6	33.1	38.6	0.139
$25,000 to $74,999	53.8	53.9	54.2	50.6	
≥ $75,000	7.5	9.6	12.7	10.9	
**Obesity Measures**^d^ **(mean ± SE)**					
BMI (kg/m^2^)	24.6 ± 4.4	24.2 ± 3.7	24.2 ± 3.3	24.5 ± 3.7	0.200
WC (cm)	81.7 ± 10.5	80.8 ± 8.8	81.7 ± 8.2	83 ± 8.8	0.010
**Alcoholic Beverage Intake (mean ± SE)**					
Total drinks/wk	0	3.4 ± 1.8	10.0 ± 2.3	25.8 ± 11.9	<0.001
Beer drinks/wk	0	2.2 ± 1.9	7 ± 3.6	18.9 ± 12.3	<0.001
Wine drinks/wk	0	0.6 ± 0.9	1.2 ± 1.9	2.3 ± 4.9	<0.001
Liquor/Mixed drinks/wk	0	0.6 ± 1.1	1.8 ± 2.3	4.5 ± 6.0	<0.001
**Dietary Intake Variables (mean ± SE)**					
Total Energy (kcal)	3220 ± 1355	3330 ± 1401	3592 ± 1440	3942 ± 1405	<0.001
Non-alcoholic Energy (kcal)^e^	3205 ± 1350	3244 ± 1391	3372 ± 1402	3477 ± 1329	0.025
Solid Food Energy (kcal)^e^	2598 ± 1104	2679 ± 1185	2812 ± 1213	2883 ± 1157	0.002
Non-alcoholic Beverage Energy (kcal)^e^	608 ± 464	565 ± 396	560 ± 374	594 ± 372	0.182
% Carb^f^	47.6 ± 7.4	44.3 ± 6.8	40.7 ± 6.7	37.3 ± 6.8	<0.001
% Prot^f^	14.7 ± 2.4	14.9 ± 2.3	14.8 ± 2.4	14.1 ± 2.4	<0.001
% Fat^f^	38.3 ± 6.1	38.5 ± 5.5	38.2 ± 5.2	36.2 ± 5.6	<0.001
Diet Quality Score	55.2 ± 11.4	57.7 ± 12	58.3 ± 11.1	57.5 ± 10.6	<0.001

^a^ Data for 1,933 men included in the analytic sample at study at exam year 0 (1985–1986) categorized according to National Institutes on Alcohol Abuse and Alcoholism guidance on drinking levels. Light drinking defined as 0> to <7 drinks/wk; moderate drinking defined as 7 to 14 drinks/wk; excessive drinking defined as >14 drinks/wk. Values are means ± SD for continuous covariates and percentages for categorical covariates. P-values are for chi^2^ tests of the unadjusted percentage distributions of categorical covariates and uncorrected overall p-value for analysis of variance (ANOVA) for means of continuous covariates. Differences were considered statistically significant at p<0.05.

^b^ Exercise Units (EU) per week.

^c^ Income was first reported at exam year 5 and values were carried backward.

^d^ Body Mass Index (BMI); Waist Circumference (WC).

^e^ Excludes energy from alcoholic beverages.

^f^ Carbohydrate contribution to total energy intake (% Carb); Protein contribution to total energy intake (% Prot); Fat contribution (% Fat) to total energy intake.

**Table 2:** Baseline characteristics of women in the CARDIA study 1985–1986, according to baseline alcoholic beverage intake (total drinks/wk)^a^.

	Total drinks/wk				
Baseline Characteristics	Non-drinker	0> to < 4	4 to 7	> 7	*p*
**N**	1,104	631	344	229	
**(%)**	47.8	27.3	14.9	9.9	
**Race (%)**					
White	38.2	52.8	60.5	72.9	<0.001
Black	61.8	47.2	39.5	27.1	
**Age cohort (%)**					
18–24 yrs	49.8	44.1	41.6	32.3	<0.001
25–30 yrs	50.2	55.9	58.4	67.7	
**Marital status (%)**					
Single, Widowed or Divorced	75.4	75.9	81.4	81.7	0.034
Married or Cohabitating	24.6	24.1	18.6	18.3	
**Smoking status (%)**					
Never smoked	68.6	58.2	41.6	31.9	<0.001
Formerly smoked	11.8	13.3	19.5	17.9	
Currently smoke	19.7	28.5	39	50.2	
**Physical Activity (EU)**^b^ **(mean ± SE)**	307.7 ± 236.4	354.2 ± 251.7	354.3 ± 249.9	404 ± 276.6	<0.001
**Education (%)**					
≤ High School	72.8	62.1	60.5	62.9	<0.001
> High School	27.2	37.9	39.5	37.1	
**Income**^c^ **(%)**					
≤ $24,999	43.4	34.2	37.8	34.1	<0.001
$25,000 to $74,999	50.5	55.9	50	50.2	
≥ $75,000	6.1	9.8	12.2	15.7	
**Obesity Measures**^d^ **(mean ± SE)**					
BMI (kg/m^2^)	24.9 ± 6	24 ± 5	24 ± 5.3	23.4 ± 4.6	<0.001
WC (cm)	74.7 ± 12	73 ± 10.5	74 ± 11.2	72.9 ± 9.7	0.009
**Alcoholic Beverage Intake (mean ± SE)**					
Total drinks/wk	0	1.8 ± 0.8	5.2 ± 1.1	14.5 ± 10.5	<0.001
Beer drinks/wk	0	0.6 ± 0.9	2.3 ± 2	7 ± 8.2	<0.001
Wine drinks/wk	0	0.8 ± 0.8	1.8 ± 1.7	3.7 ± 4.5	<0.001
Liquor/Mixed drinks/wk	0	0.4 ± 0.7	1.1 ± 1.5	3.8 ± 5.9	<0.001
**Dietary Intake Variables (mean ± SE)**					
Total Energy (kcal)	2228 ± 977	2243 ± 979	2421 ± 1010	2517 ± 979	<0.001
Non-alcoholic Energy (kcal)^e^	2219 ± 976	2209 ± 975	2338 ± 997	2331 ± 928	0.089
Solid Food Energy (kcal)^e^	1797 ± 819	1805 ± 814	1919 ± 849	1896 ± 789	0.048
Non-alcoholic Beverage Energy (kcal)^e^	422 ± 343	404 ± 294	419 ± 311	435 ± 321	0.572
% Carb^f^	48.5 ± 7.6	46.6 ± 7.2	44.3 ± 7	41.3 ± 7.8	<0.001
% Prot^f^	14.6 ± 2.9	15.1 ± 2.9	14.6 ± 2.6	14.5 ± 2.7	0.009
% Fat^f^	37.6 ± 6.3	37.4 ± 6.1	37.2 ± 6.1	35.5 ± 6.1	<0.001
Diet Quality Score	59.1 ± 12	62.8 ± 12.9	62.9 ± 12.9	64 ± 12.1	<0.001

^a^ Data for 2,308 women included in the analytic sample at study at exam year 0 (1985–1986) categorized according to National Institutes on Alcohol Abuse and Alcoholism guidance on drinking levels. Light drinking defined as 0> to < 4 drinks/wk; moderate drinking defined as 4 to 7 drinks/wk for women; excessive drinking defined as > 7 drinks/wk for women. Values are means ± SD for continuous covariates and percentages for categorical covariates. P-values for chi^2^ tests of the unadjusted percentage distributions of categorical covariates and uncorrected overall p-value for analysis of variance (ANOVA) for means of continuous covariates. Differences were considered statistically significant at p<0.05.

^b^ Exercise Units (EU) per week.

^c^ Income was first reported at exam year 5 and values were carried backward.

^d^ Body Mass Index (BMI); Waist Circumference (WC).

^e^ Excludes energy from alcoholic beverages.

^f^ Carbohydrate contribution to total energy intake (% Carb); Protein contribution to total energy intake (% Prot); Fat contribution (% Fat) to total energy intake.

### Waist circumference and body mass index gains

Within a 5-yr period, WC and BMI gains were observed across all change categories of drinking among men and women. The adjusted mean WC gain was 3.77 ± 0.18 cm and 3.78 ± 0.14 cm, among men and women with stable non-drinking, respectively. The adjusted mean BMI gain was 1.18 ± 0.06 kg/m^2^ and 1.49 ± 0.06 kg/m^2^ among men and women with stable non-drinking, respectively (S2 Table). To determine the absolute WC or BMI gain in each of the drinking change categories, the adjusted mean 5-yr WC or BMI change reported in S3 Table may be added to the adjusted mean 5-yr WC or BMI change among those who reported stable non-drinking over a 5-yr period as reported in S2 Table. The results of this computation for men and women according to 5-yr changes in total alcoholic beverage intake are reported in S2 Table.

**Fig 2:**
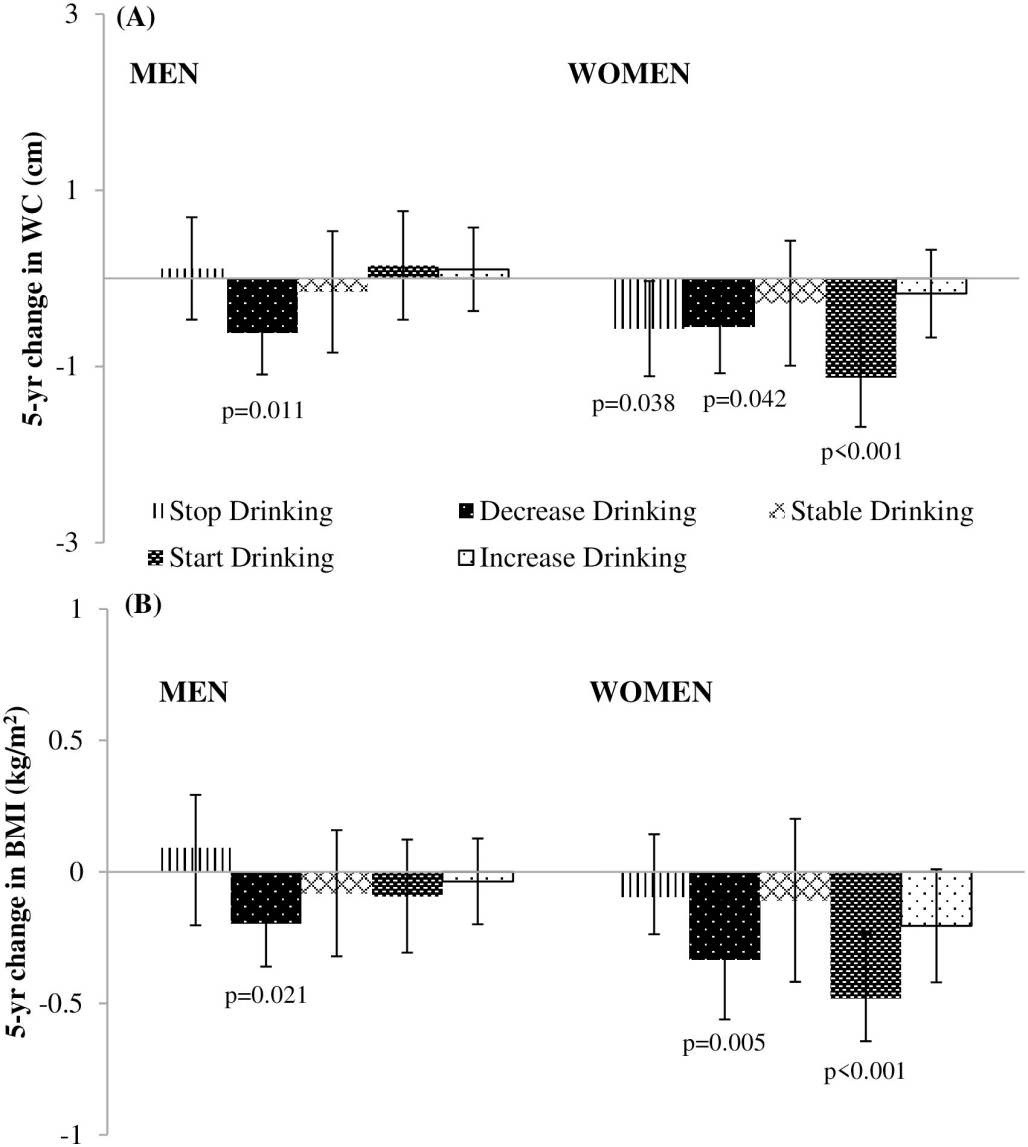
Adjusted associations of 5-yr changes in total alcoholic beverage intake with 5-yr changes in (A) Waist Circumference (WC) (cm) and (B) Body Mass Index (BMI) (kg/m^2^) for men and women in the CARDIA Study from 1985–1986 to 2010–2011. Data from men (N = 1,974) and women (N = 2,381) for 5-yr changes in **(A)** WC and **(B)** BMI from CARDIA exam yrs 5, 10, 15, 20 and 25. Values are β coefficients (95% CI) obtained from longitudinal random effects linear regression models adjusted for baseline age cohort membership, baseline WC, race and study center and time-varying income, education, smoking status and time-varying changes in marital status, physical activity and diet quality score. When 5-yr change in BMI was the outcome, models were adjusted for baseline BMI instead of baseline WC. Estimates compared to the referent 5-yr change among “stable non-drinking”. For stable non-drinking men, the adjusted mean 5-yr WC change was +3.77 ± 0.18 cm and the adjusted mean 5-yr BMI change +1.18 ± 0.06 kg/m^2^; for stable non-drinking women, the adjusted mean 5-yr WC change was +3.78 ± 0.14 cm and the adjusted mean 5-yr BMI change was +1.49 ± 0.06 kg/m^2^. P-values correspond to the 2-tailed p-values used in testing the null hypothesis that β is 0. β estimates having p-values <0.05 were considered statistically significant.

Men who decreased their total alcoholic beverage intake over a 5-yr period experienced lower 5-yr WC (β:-0.62 cm; 95% CI: -1.09, -0.14 cm (Fig 2A) and BMI gains (β:-0.20 kg/m^2^; 95% CI: -0.36, -0.03 kg/m^2^) (Fig 2B).

**Fig 3:**
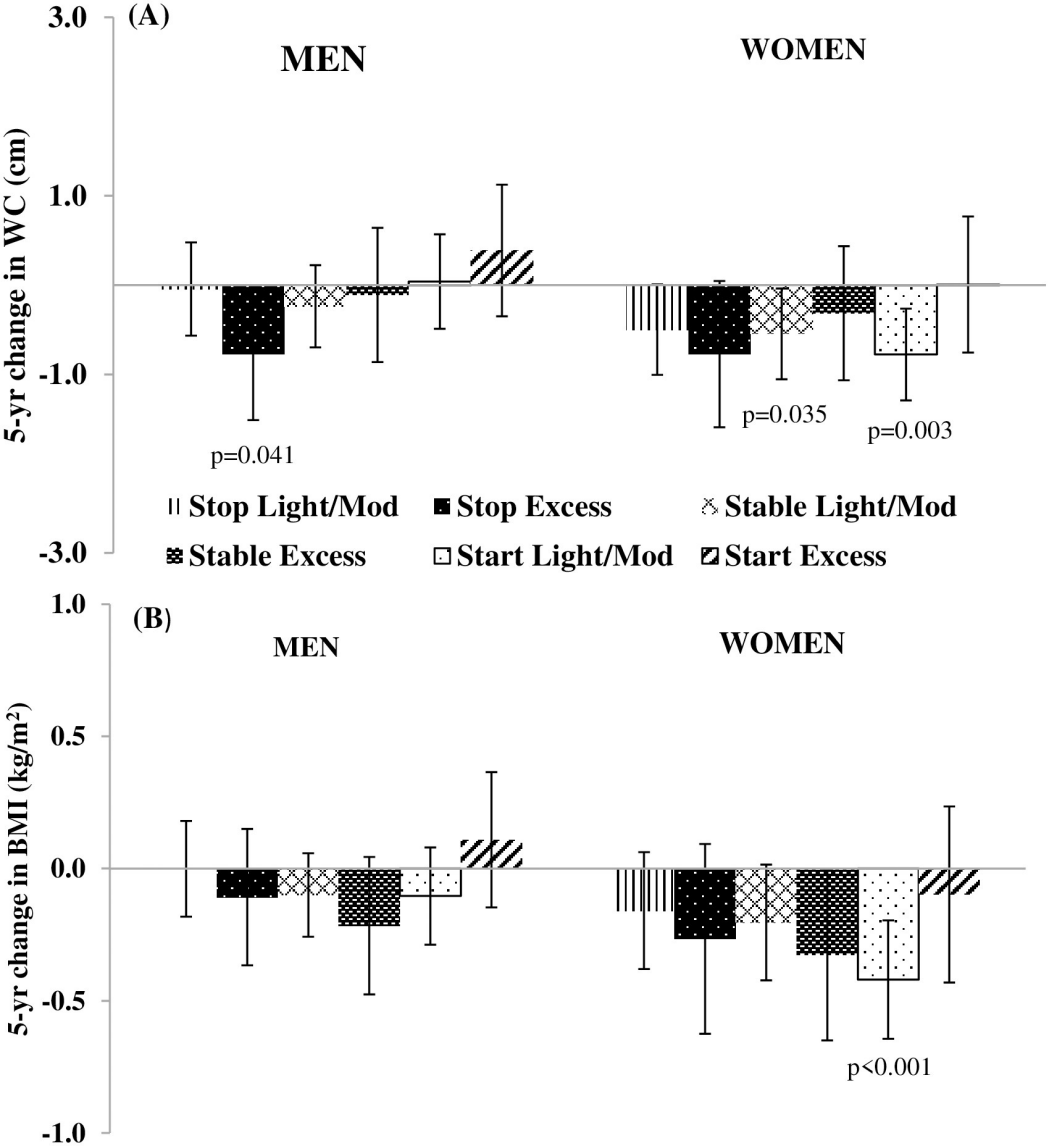
Adjusted associations of 5-yr changes in drinking level with 5-yr changes in (A) Waist Circumference (WC) (cm) and (B) Body Mass Index (BMI) (kg/m^2^) for men and women in the CARDIA Study from 1985–1986 to 2010–2011. Data from men (N = 1,974) and women (N = 2,381) for 5-yr changes in **(A)** WC and **(B)** BMI from CARDIA exam yrs 5, 10, 15, 20 and 25. Values are β coefficients (95% CI) obtained from longitudinal random effects linear regression models adjusted for baseline age cohort membership, baseline WC, race and study center and time-varying income, education, smoking status and time-varying changes in marital status, physical activity and diet quality score. When 5-yr change in BMI was the outcome, models were adjusted for baseline BMI instead of baseline WC. Estimates compared to the referent 5-yr change among “stable non-drinking”. For stable non-drinking men, the adjusted mean 5-yr WC change was +3.77 ± 0.18 cm and the adjusted mean 5-yr BMI change +1.18 ± 0.06 kg/m^2^; for stable non-drinking women, the adjusted mean 5-yr WC change was +3.78 ± 0.14 cm and the adjusted mean 5-yr BMI change was +1.49 ± 0.06 kg/m^2^. P-values correspond to the 2-tailed p-values used in testing the null hypothesis that β is 0. β estimates having p-values <0.05 were considered statistically significant.

When 5-yr changes in drinking level were examined, men who stopped excessive drinking had lower 5-yr WC gains (β:-0.77 cm; 95% CI: -1.51, -0.03 cm) (Fig 3A).

When changes in beverage type were examined, associations between 5-yr changes in beer, wine and liquor/mixed drink intakes with 5-yr WC and BMI gains were non-significant (S1 Fig) [8].

Women who started to drink over a 5-yr period experienced lower 5-yr WC (β:-1.12 cm; 95% CI: -1.69, -0.56 cm) (Fig 2A) and BMI gains (β: -0.48 kg/m^2^; 95% CI: -0.73, -0.23 kg/m^2^) (Fig 2B). Compared to stable non-drinking, lower 5-year WC and BMI gains were observed in women who decreased drinking over a 5-yr period (β: -0.55 cm; 95% CI: -1.08, -0.02cm (Fig 2A) and β:-0.33 kg/m^2^; 95% CI: -0.56, -0.10 kg/m^2^) (Fig 2B), respectively.

**Fig 4:**
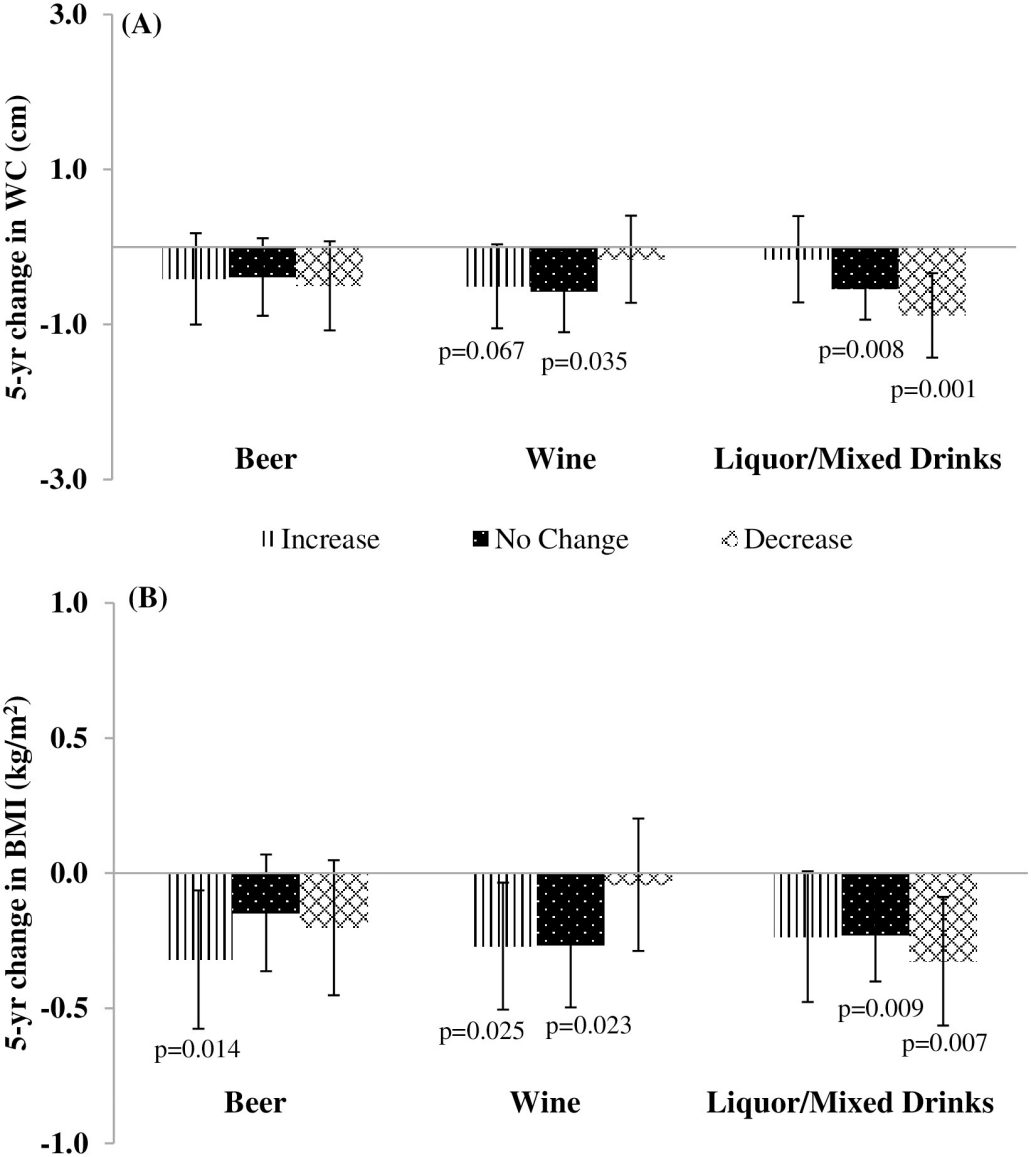
Adjusted associations of 5-yr changes in alcoholic beverage intake by type with 5-yr changes in (A) Waist Circumference (WC) (cm) and (B) Body Mass Index (BMI) (kg/m^2^) for women in the CARDIA Study from 1985–1986 to 2010–2011. Data from women (N = 2,381) for 5-yr changes in changes in **(A)** WC and **(B)** BMI from CARDIA exam yrs 5, 10, 15, 20 and 25. Values are β coefficients (95% CI) obtained from longitudinal random effects linear regression models adjusted for baseline age cohort membership, baseline WC, race and study center and time-varying income, education, smoking status and time-varying changes in marital status, physical activity, diet quality and intake of each other alcoholic beverage type. When 5-yr change in BMI was the outcome, models were adjusted for baseline BMI instead of baseline WC. Estimates compared to the referent 5-yr change among “stable non-drinking”. For stable non-drinking women, the adjusted mean 5-yr WC change was +3.78 ± 0.14 cm and the adjusted mean 5-yr BMI change was +1.49 ± 0.06 kg/m^2^. P-values correspond to the 2-tailed p-values used in testing the null hypothesis that β is 0. β estimates having p-values <0.05 were considered statistically significant.

When changes in drinking level were examined, women who started light/moderate drinking over a 5-yr period had lower 5-yr WC (β: -0.78 cm; 95% CI: -1.29, -0.26 cm) (Fig 3A) and BMI gains (β:-0.42 kg/m^2^; 95% CI: -0.64, -0.20 kg/m^2^) (Fig 3B). When 5-yr changes in beverage type were examined, a 5-yr increase in wine intake was associated with a lower 5-yr WC gain albeit with confidence intervals including the null (β: -0.51 cm; 95% CI: -1.05, 0.04 cm; p = 0.067) (Fig 4A). An increase in wine intake was also associated with lower 5-yr BMI gains (β:-0.27 kg/m^2^; 95% CI: -0.51, -0.03 kg/m^2^) (Fig 4B). Lower 5-yr WC and BMI gains were observed in women drinkers with decreasing liquor/mixed drink intake over a 5-yr period (β:-0.88 cm; 95% CI: -1.43, -0.34 cm (Fig 4A) and β:-0.33 kg/m^2^; 95% CI: -0.56, -0.09 kg/m^2^ (Fig 4B), respectively). Beer intakes were not associated with 5-yr WC change among women (Fig 4A). Yet, a 5-yr increase in beer intake was associated with lower 5-yr BMI gains (β:-0.32 kg/m^2^; 95% CI: -0.58, -0.06 kg/m^2^) (Fig 4B) [8].

### Supplemental analyses

Results were similar for 5-yr changes in total alcohol intake, drinking level and alcoholic beverage type with 5-yr changes in WC and BMI when sensitivity analyses were conducted without censoring on diabetes, hypertension or self-reported cancer (S2–S5 Figs) [8]. Including those with diabetes, hypertension or self-reported cancer had an inconsistent impact on the magnitude and statistical significance of a few estimates. Among men, the association of a 5-yr decrease in drinking with 5-yr WC change was attenuated (β:-0.52 cm; 95% CI:-0.94, -0.10 cm) (S3 Table). The association with a decrease in drinking with 5-yr BMI change in men (β: -0.14 kg/m^2^; 95% CI:-0.35, 0.07 kg/m^2^) and women (β: -0.21 kg/m^2^; 95% CI:-0.42, 0.00 kg/m^2^) was attenuated and non-significant in sensitivity analyses (S3 Table). Similarly, the association with stopping excessive drinking and WC change over a 5-yr period in men (β: -0.66 cm; 95% CI:-1.33, 0.02 cm) was attenuated and non-significant in sensitivity analyses. In women, the association of starting light/moderate drinking and WC was strengthened (β: -0.80 cm; 95% CI:-1.26 cm, -0.34 cm) and estimates of BMI change were identical (β: -0.42 kg/m^2^; 95% CI:-0.63, -0.22 kg/m^2^) in sensitivity analyses (S3 Table).

## Discussion

We observed that associations of 5-yr changes in total alcohol intake with 5-yr WC and BMI change over the 25-year study period differed between men and women and across drinking subgroups in women. In men, we found that decreasing total intake and stopping excessive consumption, were associated with lower 5-yr WC gains. In women, starting to drink, specifically starting light/moderate consumption, and increasing wine intake were associated with lower 5-yr WC and BMI gains. In contrast, decreasing total intake and decreasing liquor/mixed drink intake were also associated with lower 5-yr WC and BMI gains in women. Associations of 5-yr changes in alcoholic beverage intake by type with 5-yr WC and BMI change were observed in women but not men [8].

This study is observational and, as with all observational studies, we cannot make causal inferences. The ability to infer causation would require that all important confounders were identified, measured accurately, and included correctly in the statistical models. Even then it is likely that a secondary data analysis will suffer from residual confounding due to measurement errors [60]. For example, dietary intake was assessed at only three time points in this study and residual confounding bias by diet is likely. The amount of tobacco smoked has been associated with alcohol intake and measures of obesity [61,62]. Thus, residual confounding due to the definition of tobacco use is likely. In studies of associations of alcohol with measures of weight status smoking is typically defined as we have done (i.e., never smoked, formerly smoked, currently smoke) or by including tobacco amount based on the number of cigarettes smoked per day among those who currently smoke [22,29,55,63–67]. Prospective studies that have categorized smoking using both definitions have reported findings similar to ours [24,29,63]. In one prospective study of British men heavy drinking was associated with an increased odds of weight gain over a 5-yr period in never smokers with weaker non-significant associations observed in those who formerly smoked and those who currently smoke [24]. Similarly, in our study positive non-significant relationships with WC and BMI change were observed among men who started to drink excessively over a 5-yr period. Thus, it is possible that residual confounding bias from smoking status in our study has biased results towards the null. However, additional research is needed to understand the magnitude and direction of residual confounding bias from dietary intake and smoking status on associations of changes in alcohol intake with changes in WC and BMI. Furthermore, drinkers were identified from self-reported, weekly alcoholic beverage consumption. Thus, misclassification of drinkers due to seasonal changes in alcohol consumption, inaccurate reporting and/or infrequent consumption is possible [68]. Differential misclassification bias such as this can result in bias towards or away from the null. While underreporting of an exposure is likely to result in bias away from the null, additional research is needed to confirm this assumption. Although we cannot rule out misclassification bias of the self-reported alcoholic beverage exposure, the CARDIA Alcohol Use Questionnaire captures usual weekly drinking behavior and is less likely to misclassify participants in comparison to a shorter-term alcohol intake assessment tool [8]. Even after adjustment for multiple time-varying lifestyle and socio-demographic characteristics and controlling for time-invariant unobserved individual characteristics, conflicting findings persisted across categories of 5-yr alcohol change in relation to 5-yr WC and BMI change, particularly in women. There is a strong body of evidence indicating that ethanol metabolism, bioavailability and a dose response of alcohol’s effect on body processes differs between men and women, even after adjustment for body weight. Women have higher body fat composition and lower body water content than men of the same body weights which has been linked to differential sex-specific ethanol metabolism [69,70]. As such, it is possible that conflicting findings in women may be attributed to residual confounding from time-varying factors that cannot be measured and are related to the physiology of ethanol metabolism [59]. Residual confounding from inherent characteristics such as metabolism can bias estimates towards or away from the null. Further, it has been hypothesized that light and moderate drinkers live healthier lifestyles due to inherent immeasurable individual characteristics that might lead to lower WC and BMI gains as compared to people who don’t drink [5,25,32]. For example, light and moderate drinkers may lead more active social lives and be less isolated compared to their non-drinking and excessive drinking counterparts. Further, light and moderate drinkers have eating patterns that vary from those that drink heavily [67,71]. Even with similar total daily energy intakes, drinking heavily has been associated with lower consumption of calories from food and non-alcoholic beverages as compared to light and moderate drinking [67]. To address this bias, we used discrete interval change analyses controlling for unmeasured time-invariant characteristics associated with alcohol intake, WC, BMI, physical activity, diet quality and marital status [72,73]. Further, those who start to drink or chose not to drink may have unmeasured underlying time-varying health conditions associated with changes in WC or BMI [74]. To address this possible bias, individuals with diabetes, hypertension and self-reported cancer at baseline were excluded and individuals who developed these diseases were censored during follow-up. There is evidence suggesting that restriction to healthy individuals, as was done in the main analyses, might induce selection bias [9,75]. Selection bias specific to restricting to healthy individuals may bias results away from the null and the findings may not be generalizable to the entire study population [76]. Our results indicate that the magnitude and direction of associations of 5-yr changes in alcohol intake with 5-yr WC and BMI changes were robust to the inclusion of those with chronic diseases for almost all drink change categories. Yet, additional research is necessary to understand how restriction based on health status impacts associations of changes in alcoholic beverage consumption with changes in obesity measures [77]. A strength of this study is the sensitivity analyses conducted without censoring on disease status which serves as a comparator to the main analyses. Notably, the current analytic method assumes that associations of 5-yr changes in alcohol intake with 5-yr changes in obesity measures are constant across time and does not account for cross-sectional effects. An added strength of this study is the use of six longitudinal assessments of alcoholic beverage intake and measured anthropometric data. Multiple measurements of exposure and outcome data increase the precision of estimates [8].

In men, decreasing total weekly alcoholic beverage intake over a 5-yr period was associated with lower 5-yr WC and BMI gains. There is a paucity of literature on associations of decreasing drinking with BMI and WC change. Yet, excessive drinking, more common in men, has been associated with weight and BMI gains [70,78]. Thus, it is conceivable that decreasing intake, including stopping excessive drinking, is associated with lower WC gains for some men as we found in this study [5,78,79]. Notably, we found that stopping excessive drinking was associated with WC but not BMI change for men. This contrast suggests that the use of varying obesity measures may yield differing results across studies and contribute to inconsistent findings in the alcohol-obesity literature. To that point, a previously published study of middle-aged British men found that among those who stopped heavy drinking over a 5-yr time period, 5-yr weight gain did not differ from people who do not drink [24]. Chronic excess alcohol intake has been shown to be associated with osteopenia, as well as decreased muscle and lean mass [8,59,80,81]. In comparison to participants in the previous study, those in the current study were older and may have experienced longer exposure times to excess alcohol intake in addition to age–related changes in body composition [82]. These physiological conditions may have differential impacts on associations of alcohol intake with weight status, overall body size (BMI), and WC. Specifically, an interaction of age and excessive drinking might lead to inverse associations among older adults and positive and/or null associations among younger adults. Future prospective research examining changes in excessive drinking levels, particularly excess intake modified by age, in relation to changes in obesity measures with consistent exposure definitions and standardized outcomes are needed to establish the evidence base [8,83–85].

Light and moderate drinking, more common in women, has been associated with the prevention of weight gain [5,78,79]. Starting to consume alcohol and starting light/moderate drinking, were associated with lower 5-yr WC and BMI gains for women in our study. Similarly, increasing alcohol intake up to moderate daily levels over an 8-yr period has been associated with lower 8-yr weight gain in US women [23]. We also found that in women who drink increasing wine intake over a 5-yr period was associated with lower BMI/WC gains as compared to stable non-drinking. In comparison, lower drinking level increases in wine intake (< 0.5 servings per day) have been associated with lower 4-yr weight gain; whereas higher drinking level increases (> 1 serving per day) have been associated with greater 4-yr weight gain among women in the US [29]. The polyphenolic compounds in red wine (i.e., resveratrol) may have beneficial effects on lipid metabolism which might lead to lower overall adiposity accumulation reflected in lower BMI/WC gains among those who drink [5,25,79,86–88]. Thus, light to moderate drinking patterns coupled with polyphenols may contribute to lower WC and BMI gains in women who start to drink as compared to stable non-drinking; however additional research is needed to substantiate this hypothesis [8].

In contrast to findings that starting to consume alcohol and increasing wine intake were associated with lower BMI/WC gains, we found that decreasing total alcohol intake, particularly decreasing liquor/mixed drink intake, was associated with lower 5-yr WC and BMI gains in women. Compared to those with no change in intake, decreasing liquor intake has been associated with lower 4-yr weight gain in women [29]. Our findings add to previous reports that liquor consumption may contribute to increases in obesity-related outcomes over time [25]. In a 2004 study of Danish men and women, compared to those who did not drink, women who drank ≥ 4 drinks/wk of beer or spirits had higher subsequent 6-year WC changes [89]. In a later study using follow-up data from the same cohort, spirit consumption was positively associated with 5-yr WC change in women [90]. A standard drink in the US is the equivalent to a twelve ounce can of beer that contains 150 calories (5%ABV), 5 ounces of wine that contains 120 calories (12% ABV), 1.5 ounces of liquor that contains 100 calories (40%ABV), a rum and cola with 190 calories (40% ABV) and a 4.5 oz piña colada cocktail that can contain as much as 245 calories [25,27,91]. Liquor with or without a mixer has a higher %ABV and can contain almost twice the calories of a drink of beer or wine. Additionally, alcohol itself is an energy-dense, nutritionally poor, substance with an energy content of 7.1 calories per gram. However, alcohol metabolism differs from other nutrients. For example, the thermogenic effect of alcohol is much greater than that of carbohydrates and fat. Making it difficult to draw direct conclusions about how alcohol itself impacts energy balance [25]. Yet, independent of the alcohol itself, the high calorie content of a mixed drink could contribute to weight and WC gain if consumed in excess to normal dietary intake. Furthermore, liquor drinking has been associated with excessive drinking in the US [12]. It could be the case that decreasing liquor/mixed drink intake subsequent to excessive levels of consumption might contribute to the management of WC and BMI gains in women who drink. Relationships of changes in alcoholic beverage type with changes in WC and BMI are complex. Future research should focus on elucidating interactions of drinking level and alcoholic beverage type in associations of changes in alcohol intake with WC and BMI change among women [8].

Similar to our study, reported differences in obesity-related outcomes between those who drink and those who do not are generally small [23,92–94]. While the magnitude of associations of changes in alcoholic beverage intake with changes in WC and BMI may be statistically small, effect sizes between 0.30 and 0.50 have been considered minimally clinically meaningful for some patient self-reported health outcomes [95,96]. Further, our estimates suggest that sustained reductions in alcohol intake could equate to lower WC and BMI gains with aging. For example, among men starting to drink was associated with a 3.92 cm 5-yr WC gain. Over a 15-year period (three 5-year intervals), starting to drink could lead to as much as a 58.8 cm gain compared to a 47.4 cm gain among men who decrease drinking. Consequently changes in alcohol intake could have large impacts on obesity measures at individual and population levels over time [97]. The results of our study could be considered with DGA guidance in the development of diet and alcohol-related public health messages specific to aging [98].

Furthermore, our findings have policy relevance. The DGA recommends persons who do not drink should not start drinking and that alcoholic beverages be consumed in moderate amounts of no more than 1 drink per day for women and no more than 2 drinks per day for men. The DGA specifically states that among adults who choose to drink, average energy intake from alcoholic beverages exceeds the remaining calorie limit that is available after food group recommendations are met [4]. Recent studies have highlighted increasing trends in calories consumed from alcoholic beverages and in drinking that exceeds moderate amounts in the US [3,74]. According to the DGA, among those who drink, alcoholic beverages contribute 9% to total caloric intake. On the days that drinking occurs, alcoholic beverage consumption typically exceeds moderate amounts and regular consumption of alcoholic beverages likely leads to the consumption of excess calories [4]. Thus, reductions in calories from alcohol intake could have large impacts on obesity measures at individual and population levels [97]. However, public knowledge of the DGA drinking guidelines and the extent to which concerns related to excess energy intake might motivate changed drinking behavior is unknown. Future research aimed at understanding consumer knowledge of moderate drinking guidelines and the extent to which calorie-related concerns might motivate changed drinking behavior is needed.

## Conclusions

The magnitude of associations of alcohol change with WC and BMI changes was small but could be clinically meaningful for both sexes in our study. The current study is one of the first to examine changes in WC and BMI in relation to changes in alcoholic beverage consumption by drinking level and beverage type in a US-based cohort. These findings add to previous reports that light and moderate drinking, wine intake, and decreasing liquor/mixed drink intake are associated with WC and BMI change in women. In men decreasing total alcohol intake, with an emphasis on stopping excessive drinking may be may be beneficial in managing WC and BMI gains [8]. Public health nutrition interventions that emphasize reductions in alcohol intake as part of dietary changes are needed to inform weight-related health care recommendations and policy.

## Supporting information

S1 Fig Adjusted associations of 5-year changes in alcoholic beverage intake by type with 5-year changes in (A) Waist Circumference (WC) (cm) and (B) Body Mass Index (BMI) in men in the CARDIA Study. Data from men (N = 1,974) for 5-year changes in WC from CARDIA exam years 5, 10, 15, 20 and 25. Values are β coefficients (95% CI) obtained from longitudinal random effects linear regression models adjusted for baseline age cohort membership, baseline WC, race and study center and time-varying income, education, smoking status and time-varying changes in marital status, physical activity, diet quality and intake of each other alcoholic beverage type. When 5-yr change in BMI was the outcome, models were adjusted for baseline BMI instead of baseline WC. Estimates compared to the referent 5-yr change among “stable non-drinking”. P-values correspond to the 2-tailed p-values used in testing the null hypothesis that β is 0. β estimates having p-values <0.05. were considered statistically significant.
(TIF)

S2 Fig Adjusted associations of 5-year changes in total alcoholic beverage intake with 5-year changes in (A) Waist Circumference (WC) (cm) and (B) Body Mass Index (BMI) (kg/m2) for men and women in the CARDIA Study without censoring for disease status Data from men (N = 1,984) and women (N = 2,393) for 5-year changes in (A) WC and (B) BMI from CARDIA exam years 5, 10, 15, 20 and 25. Values are β coefficients (95% CI) obtained from longitudinal random effects linear regression models adjusted for baseline age cohort membership, baseline WC, race and study center and time-varying income, education, smoking status and time-varying changes in marital status, physical activity and diet quality score. When 5-yr change in BMI was the outcome, models were adjusted for baseline BMI instead of baseline WC. Estimates compared to the referent 5-yr change among “stable non-drinking”. P-values correspond to the 2-tailed p-values used in testing the null hypothesis that β is 0. Β estimates having p-values <0.05. were considered statistically significant.
(TIF)

S3 Fig Adjusted associations of 5-year changes in drinking level with 5-year changes in (A) Waist Circumference (WC) (cm) and (B) Body Mass Index (BMI) (kg/m2) for men and women in the CARDIA Study without censoring for disease status. Data from men (N = 1,984) and women (N = 2,393) for 5-year changes in (A) WC and (B) BMI from CARDIA exam years 5, 10, 15, 20 and 25. Values are β coefficients (95% CI) obtained from longitudinal random effects linear regression models adjusted for baseline age cohort membership, baseline WC, race and study center and time-varying income, education, smoking status and time-varying changes in marital status, physical activity and diet quality score. When 5-yr change in BMI was the outcome, models were adjusted for baseline BMI instead of baseline WC. Estimates compared to the referent 5-yr change among “stable non-drinking”. P-values correspond to the 2-tailed p-values used in testing the null hypothesis that β is 0. β estimates having p-values <0.05. were considered statistically significant.
(TIF)

S4 Fig Adjusted associations in women of 5-year changes in alcoholic beverage intake by type with 5-year changes in (A) Waist Circumference (WC) (cm) and (B) Body Mass Index (BMI) (kg/m2) for women in the CARDIA Study without censoring for disease status. Data from women (N = 2,393) for 5-year changes in BMI from CARDIA exam years 5, 10, 15, 20 and 25. Values are β coefficients (95% CI) obtained from longitudinal random effects linear regression models adjusted for baseline age cohort membership, baseline WC, race and study center and time-varying income, education, smoking status and time-varying changes in marital status, physical activity, diet quality and intake of each other alcoholic beverage type. When 5-yr change in BMI was the outcome, models were adjusted for baseline BMI instead of baseline WC. Estimates compared to the referent 5-yr change among “stable non-drinking”. P-values correspond to the 2-tailed p-values used in testing the null hypothesis that β is 0. β having p-values <0.05. were considered statistically significant.
(TIF)

S5 Fig Adjusted associations in men of 5-year changes in alcoholic beverage intake by type with 5-year changes in (A) Waist Circumference (WC) (cm) and (B) Body Mass Index (BMI) in men in the CARDIA Study without censoring for disease status. Data from men (N = 1,984) for 5-year changes in WC from CARDIA exam years 5, 10, 15, 20 and 25. Values are β coefficients (95% CI) obtained from longitudinal random effects linear regression models adjusted for baseline age cohort membership, baseline WC, race and study center and time-varying income, education, smoking status and time-varying changes in marital status, physical activity, diet quality and intake of each other alcoholic beverage type When 5-yr change in BMI was the outcome, models were adjusted for baseline BMI instead of baseline WC. Estimates compared to the referent 5-yr change among “stable non-drinking”. P-values correspond to the 2-tailed p-values used in testing the null hypothesis that β is 0. β estimates having p-values <0.05. were considered statistically significant.
(TIF)

S1 Table Distribution of select baseline characteristics of individuals according to inclusion and exclusion status. Data for 5,115 men and women included in the CARDIA study at baseline, minus one enrolled participant who dropped out. Values are percentages unless N specified. Sample sizes vary for baseline covariates because the data are an unbalanced panel with some participant observations missing at baseline and included in future waves. P-values for chi2 tests of the unadjusted percentage distributions of categorical covariates of included individuals compared to the percentage distribution of covariates of excluded individuals. Differences were considered statistically significant at p<0.05. Beer, wine, and liquor/mixed drinks intake were categorized according to National Institutes on Alcohol Abuse and Alcoholism guidance on drinking levels. Light drinking defined as 0> to <7 drinks/wk; moderate drinking defined as 7 to 14 drinks/wk; excessive drinking defined as >14 drinks/wk for men. Light drinking defined as 0> to < 4 drinks/wk; moderate drinking defined as 4 to 7 drinks/wk for women; excessive drinking defined as > 7 drinks/wk for women. Abdominal obesity was defined as waist circumference (WC) >102 cm for men and >88 cm for women.
(DOCX)

S2 Table Adjusted mean 5-yr WC and BMI changes for men and women in the CARDIA Study from 1985–1986 to 2010–2011. Data from men (N = 1,974) and women (N = 2,381) for adjusted mean 5-yr changes and standard errors (SE) in WC and BMI from CARDIA exam yrs 5, 10, 15, 20 and 25. Values are the adjusted mean 5-yr changes, SE and 95% confidence intervals (95% CI) obtained from longitudinal random effects linear regression models adjusted for baseline age cohort membership, baseline WC, race and study center and time-varying income, education, smoking status and time-varying changes in marital status, physical activity and diet quality score. When 5-yr change in BMI was the outcome, models were adjusted for baseline BMI instead of baseline WC.
(DOCX)

S3 Table Adjusted associations of 5-yr changes in alcoholic beverage intake with 5-yr changes in (A) Waist Circumference (WC) (cm) and (B) Body Mass Index (BMI) (kg/m^2^) for men and women in the CARDIA Study from 1985–1986 to 2010–2011. Values are β coefficients and 95% confidence intervals (95% CI) obtained from longitudinal random effects linear regression models. ^a^ Data from men (N = 1,974) and women (N = 2,381) with observations censored for participants with diabetes, hypertension or self-reported cancer during follow-up at the year in which the disease was reported. ^b^ Data from men (N = 1,984) and women (N = 2,393) without censoring observations on diabetes, hypertension or self-reported cancer. ^c^ Model adjusted for baseline age cohort membership, baseline WC, race and study center and time-varying income, education, smoking status and time-varying changes in marital status, physical activity, and diet quality score. When 5-yr change in BMI was the outcome, models were adjusted for baseline BMI instead of baseline WC. ^d^ Model adjusted for baseline age cohort membership, baseline WC, race and study center and time-varying income, education, smoking status and time-varying changes in marital status, physical activity, diet quality and intake of each other alcoholic beverage type. When 5-yr change in BMI was the outcome, models were adjusted for baseline BMI instead of baseline WC.
(DOCX)

S1 File Supplemental methods and analyses. A secondary analytic dataset, that did not include censoring on diabetes, hypertension or self-reported cancer, was created. This secondary dataset included six CARDIA exams (1985–1986, 1990–1991, 1995–1996, 2000–2001, 2005–2006, 2010–2011). All adults with socio-demographic data at baseline were considered eligible participants excluding one participant who withdrew from the study (N = 5,114). As has been done in previous studies, to minimize bias resulting from illness that may affect body weight, we excluded participants with hypertension (SBP/DBP ≥ 140/≥90 mm Hg or taking medication for elevated BP; N = 145) diabetes (fasting glucose ≥126 mg/dL or taking medication for diabetes; N = 29) or cancer (self-reported diagnosis; N = 137) at baseline. (29,37) Further, we excluded participants who were missing data on diabetes, hypertension, self-reported cancer diagnoses (N = 9) at each exam and those missing waist circumference (N = 17) or BMI (N = 4) at exam year 0. We also excluded participants who attended only one of the six exams used in the current study exam (N = 351). Individuals with excluded observations at every exam were excluded from the analytic sample (N = 45). For individuals included in the analytic sample, observations were excluded at given exam years if participants were pregnant or breastfeeding (observations = 305) or had implausible energy intakes (<600 kcal/d or >6000/d kcal for women and <800 kcal/d or >8000 kcal/d for men) (observations = 393) at an exam or if they were missing exposure (observations = 1347 alcohol intake), outcome (observations = 34 WC, 131 BMI), or covariate data at a given exam year (observations = 1 education, 6 marital status, 102 smoking, 50 physical activity, 1,159 dietary intake). Our final analytic sample consisted of 4,377 participants (men and women) n = 4,241 at year 0; 3,771 at year 5; 2,966 at year 10; 2,717 at year 15; 2,513 at year 20 and 2,463 at year 25 for a total 18,671 person observations [8].
(DOCX)
