# Supplementary figures and images for: Associations of 5-year changes in alcoholic beverage intake with 5-year changes in waist circumference and BMI in the Coronary Artery Risk Development in Young Adults (CARDIA) study
Source: PLoS One. 2023 Mar 8;18(3):e0281722. doi: 10.1371/journal.pone.0281722 (PMC9994756; doi:10.1371/journal.pone.0281722)

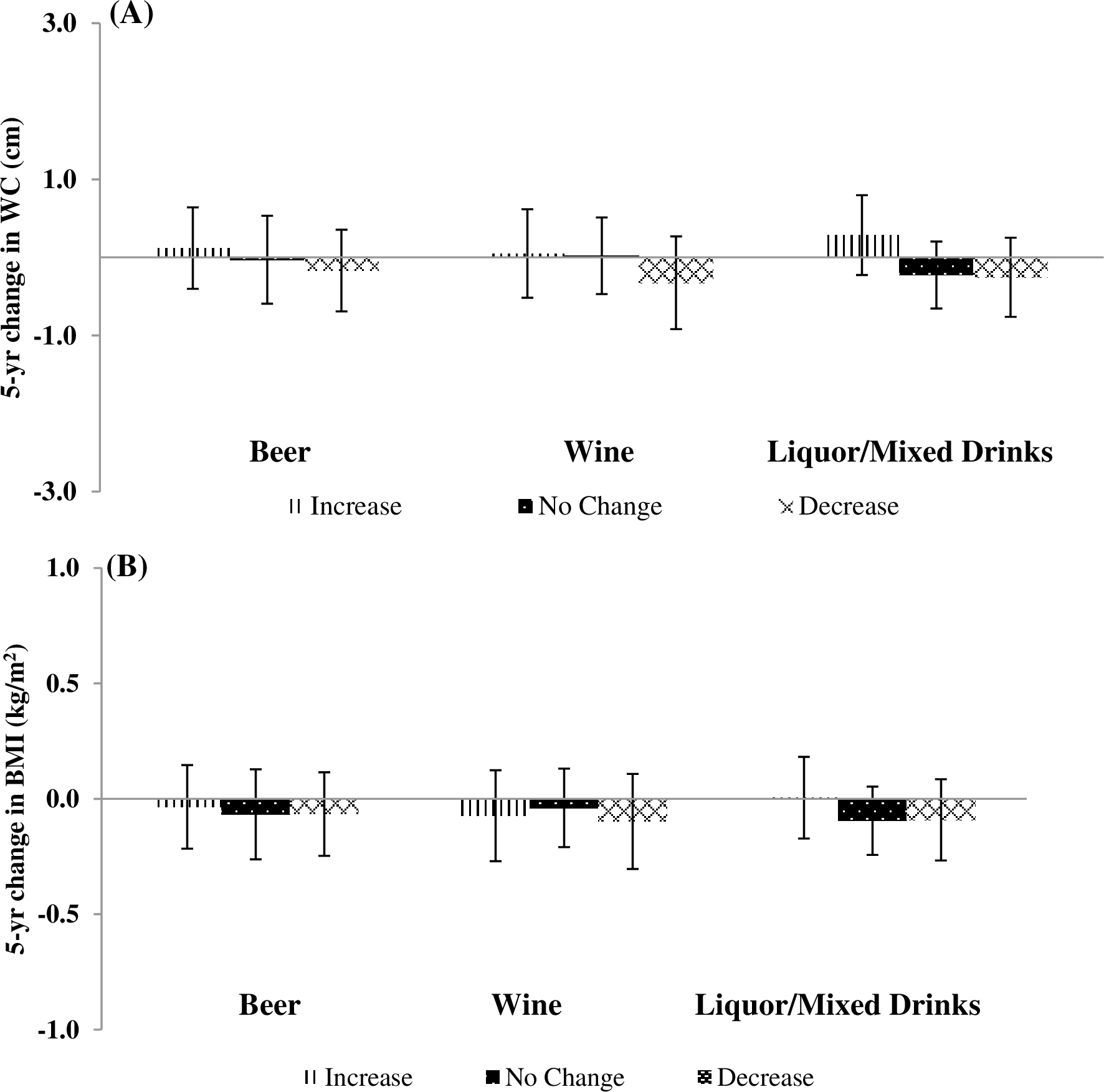

Supplement: S1 Fig — Data from men (N = 1,974) for 5-year changes in WC from CARDIA exam years 5, 10, 15, 20 and 25. Values are β coefficients (95% CI) obtained from longitudinal random effects linear regression models adjusted for baseline age cohort membership, baseline WC, race and study center and time-varying income, education, smoking status and time-varying changes in marital status, physical activity, diet quality and intake of each other alcoholic beverage type. When 5-yr change in BMI was the outcome, models were adjusted for baseline BMI instead of baseline WC. Estimates compared to the referent 5-yr change among “stable non-drinking”. P-values correspond to the 2-tailed p-values used in testing the null hypothesis that β is 0. β estimates having p-values <0.05. were considered statistically significant. (TIF) [file pone.0281722.s001.tif]

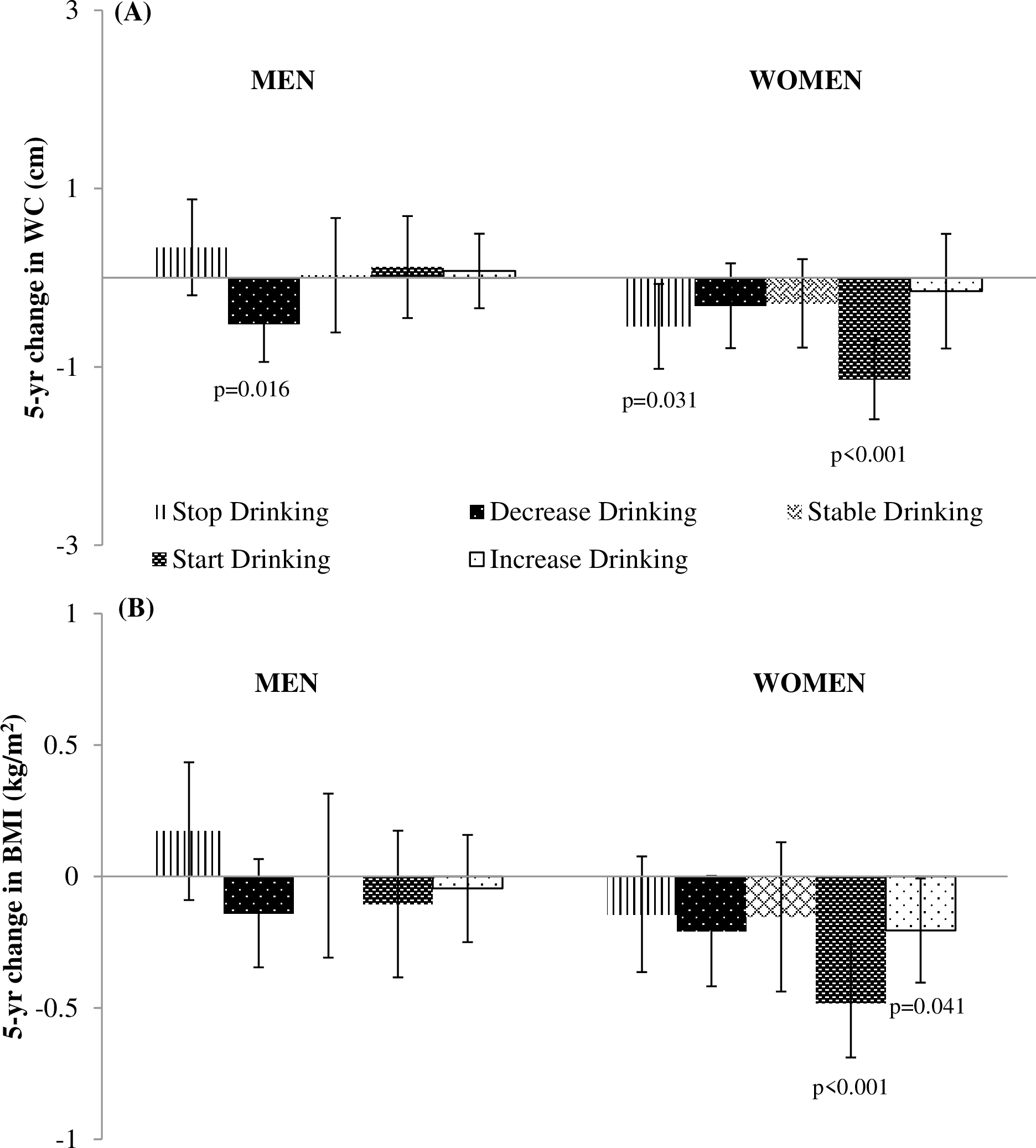

Supplement: S2 Fig — Values are β coefficients (95% CI) obtained from longitudinal random effects linear regression models adjusted for baseline age cohort membership, baseline WC, race and study center and time-varying income, education, smoking status and time-varying changes in marital status, physical activity and diet quality score. When 5-yr change in BMI was the outcome, models were adjusted for baseline BMI instead of baseline WC. Estimates compared to the referent 5-yr change among “stable non-drinking”. P-values correspond to the 2-tailed p-values used in testing the null hypothesis that β is 0. Β estimates having p-values <0.05. were considered statistically significant. (TIF) [file pone.0281722.s002.tif]

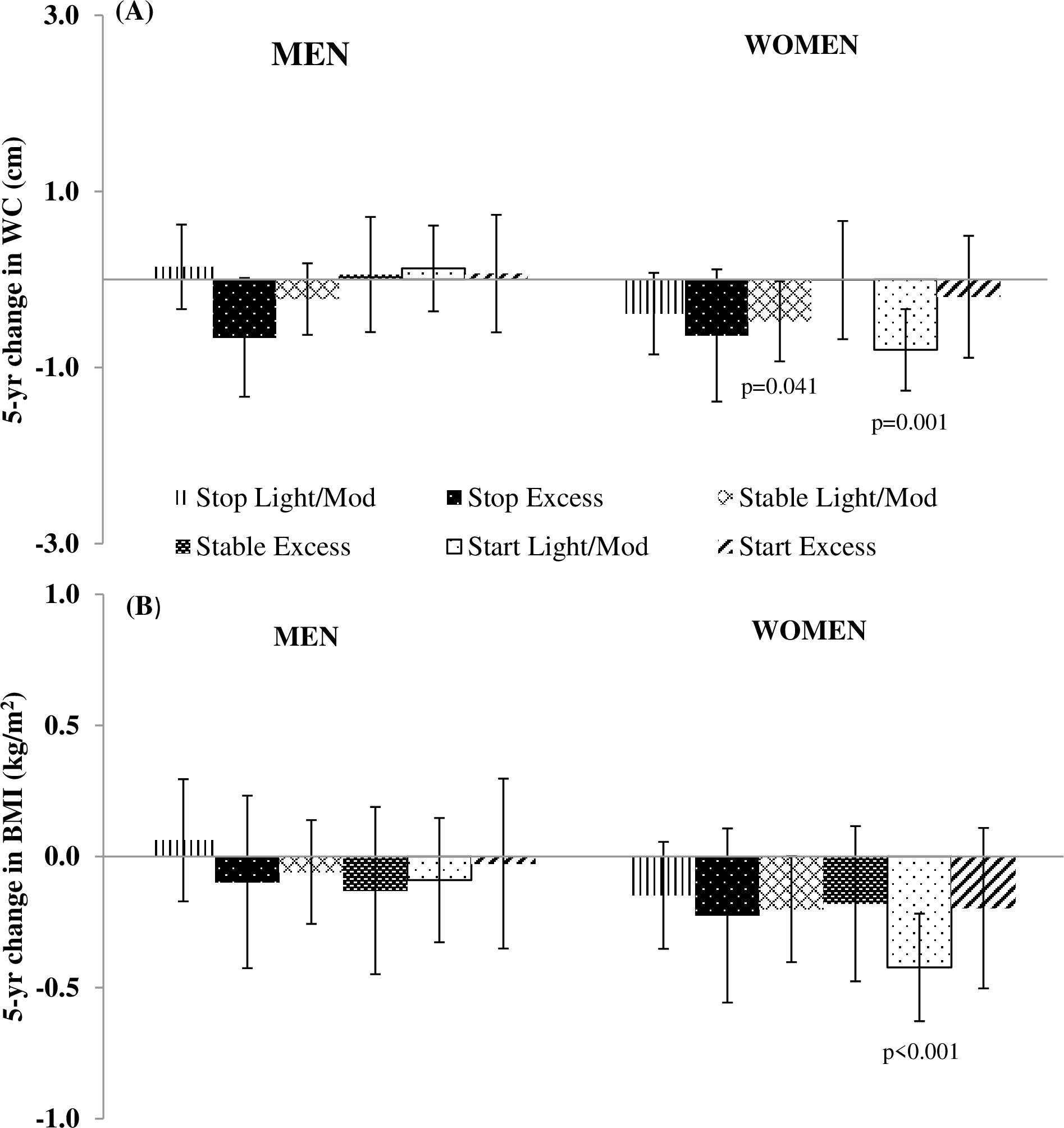

Supplement: S3 Fig — Data from men (N = 1,984) and women (N = 2,393) for 5-year changes in (A) WC and (B) BMI from CARDIA exam years 5, 10, 15, 20 and 25. Values are β coefficients (95% CI) obtained from longitudinal random effects linear regression models adjusted for baseline age cohort membership, baseline WC, race and study center and time-varying income, education, smoking status and time-varying changes in marital status, physical activity and diet quality score. When 5-yr change in BMI was the outcome, models were adjusted for baseline BMI instead of baseline WC. Estimates compared to the referent 5-yr change among “stable non-drinking”. P-values correspond to the 2-tailed p-values used in testing the null hypothesis that β is 0. β estimates having p-values <0.05. were considered statistically significant. (TIF) [file pone.0281722.s003.tif]

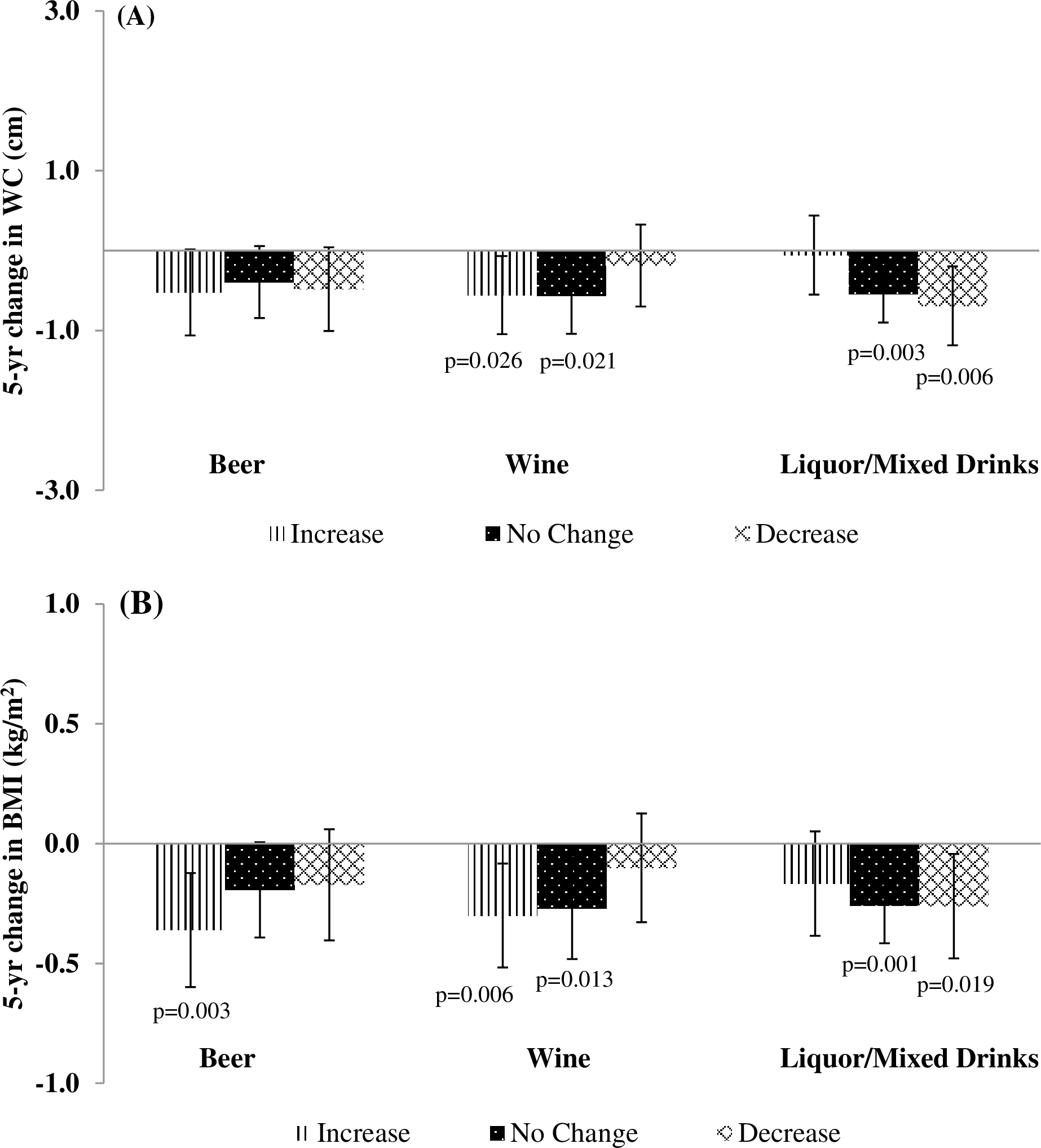

Supplement: S4 Fig — Data from women (N = 2,393) for 5-year changes in BMI from CARDIA exam years 5, 10, 15, 20 and 25. Values are β coefficients (95% CI) obtained from longitudinal random effects linear regression models adjusted for baseline age cohort membership, baseline WC, race and study center and time-varying income, education, smoking status and time-varying changes in marital status, physical activity, diet quality and intake of each other alcoholic beverage type. When 5-yr change in BMI was the outcome, models were adjusted for baseline BMI instead of baseline WC. Estimates compared to the referent 5-yr change among “stable non-drinking”. P-values correspond to the 2-tailed p-values used in testing the null hypothesis that β is 0. β having p-values <0.05. were considered statistically significant. (TIF) [file pone.0281722.s004.tif]

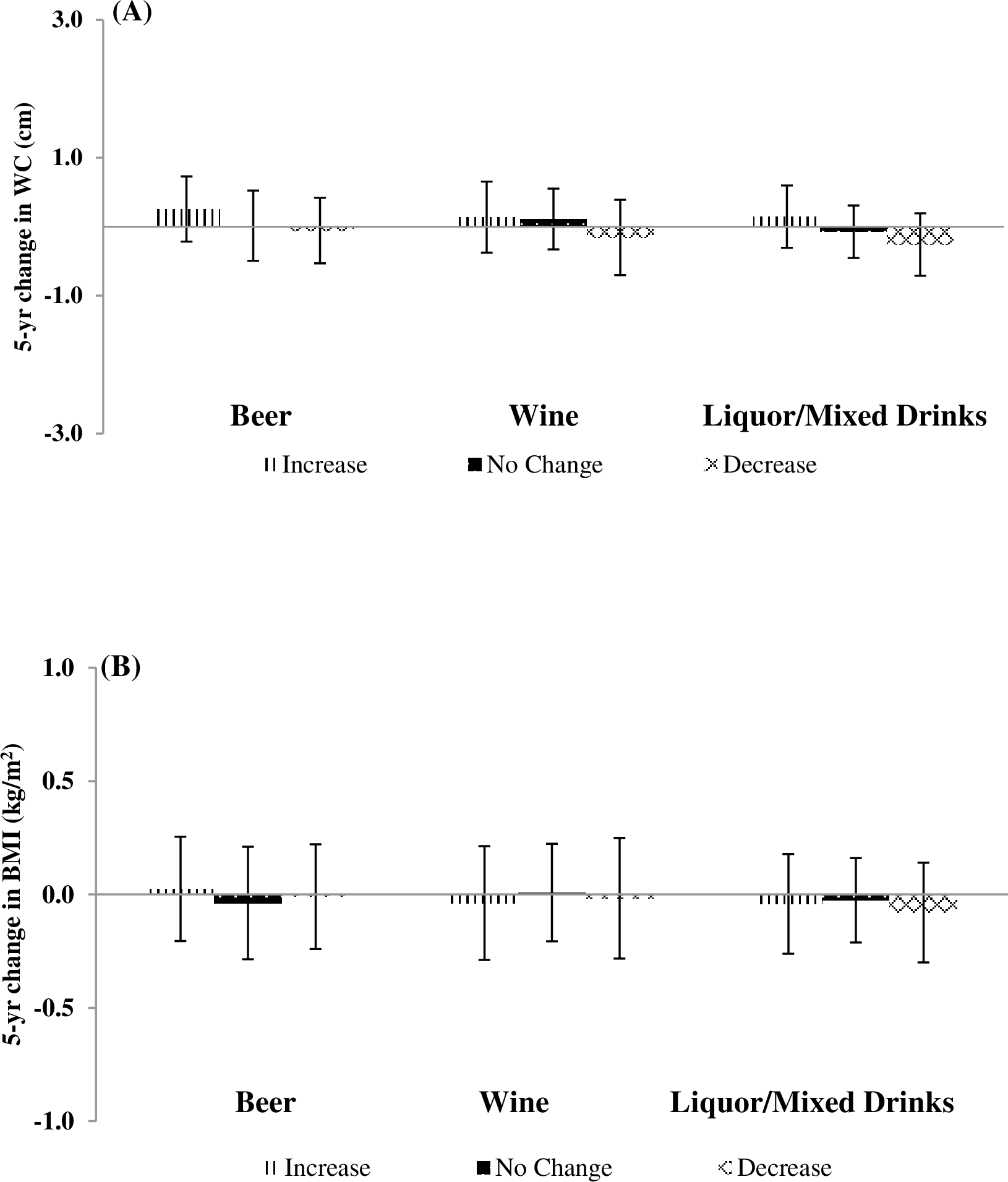

Supplement: S5 Fig — Data from men (N = 1,984) for 5-year changes in WC from CARDIA exam years 5, 10, 15, 20 and 25. Values are β coefficients (95% CI) obtained from longitudinal random effects linear regression models adjusted for baseline age cohort membership, baseline WC, race and study center and time-varying income, education, smoking status and time-varying changes in marital status, physical activity, diet quality and intake of each other alcoholic beverage type When 5-yr change in BMI was the outcome, models were adjusted for baseline BMI instead of baseline WC. Estimates compared to the referent 5-yr change among “stable non-drinking”. P-values correspond to the 2-tailed p-values used in testing the null hypothesis that β is 0. β estimates having p-values <0.05. were considered statistically significant. (TIF) [file pone.0281722.s005.tif]
