# Supplementary material for: Associations of 5-year changes in alcoholic beverage intake with 5-year changes in waist circumference and BMI in the Coronary Artery Risk Development in Young Adults (CARDIA) study
Source: PLoS One. 2023 Mar 8;18(3):e0281722. doi: 10.1371/journal.pone.0281722 (PMC9994756; doi:10.1371/journal.pone.0281722)
Supplement: S1 Table — Data for 5,115 men and women included in the CARDIA study at baseline, minus one enrolled participant who dropped out. Values are percentages unless N specified. Sample sizes vary for baseline covariates because the data are an unbalanced panel with some participant observations missing at baseline and included in future waves. P-values for chi2 tests of the unadjusted percentage distributions of categorical covariates of included individuals compared to the percentage distribution of covariates of excluded individuals. Differences were considered statistically significant at p<0.05. Beer, wine, and liquor/mixed drinks intake were categorized according to National Institutes on Alcohol Abuse and Alcoholism guidance on drinking levels. Light drinking defined as 0> to <7 drinks/wk; moderate drinking defined as 7 to 14 drinks/wk; excessive drinking defined as >14 drinks/wk for men. Light drinking defined as 0> to < 4 drinks/wk; moderate drinking defined as 4 to 7 drinks/wk for women; excessive drinking defined as > 7 drinks/wk for women. Abdominal obesity was defined as waist circumference (WC) >102 cm for men and >88 cm for women. (DOCX) [file pone.0281722.s006.docx]

|  | **Included** | **Excluded** | **p-value** |
| --- | --- | --- | --- |
| **N** | 4,355 | 759 |  |
| **Sex (N)** | 4,355 | 759 |  |
| Female (%) | 54.7 | 53.5 | 0.546 |
| Male (%) | 45.3 | 46.5 |  |
| **Race (N)** | 4,355 | 759 |  |
| White (%) | 50.4 | 36.9 | <0.001 |
| Black (%) | 49.6 | 63.1 |  |
| **Age cohort at baseline (N)** | 4,355 | 759 |  |
| 17-24 yrs (%) | 45.1 | 42 | 0.122 |
| 25-30 yrs (%) | 54.9 | 58 |  |
| **Education (N)** | 4,353 | 758 |  |
| ≤ High School (%) | 67.4 | 73.0 | 0.003 |
| > High School (%) | 32.6 | 27.0 |  |
| **Marital Status (N)** | 4,353 | 755 |  |
| Single/widowed/divorced (%) | 77.7 | 78.0 | 0.835 |
| Married/co-habitating (%) | 22.3 | 22.0 |  |
| **Smoking status (N)** | 4,332 | 746 |  |
| Never Smoked (%) | 57.2 | 51.0 | <0.001 |
| Formerly Smoked (%) | 13.6 | 11.5 |  |
| Currently Smokes (%) | 29.2 | 37.5 |  |
| **Total drinks/wk (N)** | 4,354 | 757 |  |
| Do Not Drink (%) | 39.2 | 37.5 | 0.009 |
| Drink Lightly (%) | 31.3 | 27.9 |  |
| Drink Moderately (%) | 17.7 | 18.9 |  |
| Drink Excessively (%) | 11.8 | 15.7 |  |
| **Beer drinks/wk (N)** | 4,354 | 757 |  |
| Do Not Drink (%) | 39.2 | 37.5 | 0.324 |
| Drink Lightly (%) | 29.8 | 28.8 |  |
| Drink Moderately (%) | 10 | 11.8 |  |
| Drink Excessively (%) | 5.7 | 7 |  |
| Drinker – no beer (%) | 15.3 | 14.9 |  |
| **Wine drinks/wk (N)** | 4,354 | 757 |  |
| Drink Lightly (%) | 39.2 | 37.5 | 0.005 |
| Drink Moderately (%) | 25.6 | 22.9 |  |
| Drink Excessively (%) | 3.6 | 6.2 |  |
| Drink Lightly (%) | 1.1 | 1.3 |  |
| Drinker – no wine (%) | 30.6 | 32.1 |  |
| **Liquor/Mixed drinks/wk (N)** | 4,354 | 757 |  |
| Drink Lightly (%) | 39.2 | 37.5 | 0.354 |
| Drink Moderately (%) | 23.8 | 24.2 |  |
| Drink Excessively (%) | 3 | 3.7 |  |
| Drink Lightly (%) | 1.4 | 2.2 |  |
| Drinker - no liquor/mixed drinks (%) | 32.6 | 32.4 |  |
| **Baseline Weight Status (kg/m^2^)** | 4,355 | 742 |  |
| Body Mass Index < 25.0 (%) | 66.1 | 60.8 | 0.001 |
| Body Mass Index ≥ 25 to < 30 (%) | 22.9 | 23.5 |  |
| Body Mass Index ≥30 (%) | 11.0 | 15.8 |  |
| **Baseline Abdominal Obesity** | 4,355 | 739 |  |
| No (%) | 92.7 | 88.1 | <0.001 |
| Yes (%) | 7.3 | 11.9 |  |
