# Supplementary material for: Associations of 5-year changes in alcoholic beverage intake with 5-year changes in waist circumference and BMI in the Coronary Artery Risk Development in Young Adults (CARDIA) study
Source: PLoS One. 2023 Mar 8;18(3):e0281722. doi: 10.1371/journal.pone.0281722 (PMC9994756; doi:10.1371/journal.pone.0281722)
Supplement: S2 Table — Data from men (N = 1,974) and women (N = 2,381) for adjusted mean 5-yr changes and standard errors (SE) in WC and BMI from CARDIA exam yrs 5, 10, 15, 20 and 25. Values are the adjusted mean 5-yr changes, SE and 95% confidence intervals (95% CI) obtained from longitudinal random effects linear regression models adjusted for baseline age cohort membership, baseline WC, race and study center and time-varying income, education, smoking status and time-varying changes in marital status, physical activity and diet quality score. When 5-yr change in BMI was the outcome, models were adjusted for baseline BMI instead of baseline WC. (DOCX) [file pone.0281722.s007.docx]

| **Men** | | |  |
| --- | --- | --- | --- |
| **WC** | Mean (cm) | ± SE (cm) | 95% CI |
| Stable non-drinking | 3.77 | 0.18 | (3.43, 4.12) |
| Start drinking | 3.92 | 0.27 | (3.40, 4.45) |
| Increase drinking | 3.88 | 0.16 | (3.56, 4.2) |
| Stable drinking | 3.62 | 0.31 | (3.02, 4.22) |
| Stop drinking | 3.89 | 0.25 | (3.41, 4.37) |
| Decrease drinking | 3.16 | 0.16 | (2.84, 3.48) |
| **BMI** | Mean (kg/m^2^) | ± SE (kg/m^2^) | 95% CI |
| Stable non-drinking | 1.18 | 0.06 | (1.06, 1.3) |
| Start drinking | 1.09 | 0.09 | (0.90, 1.27) |
| Increase drinking | 1.14 | 0.06 | (1.03, 1.25) |
| Stable drinking | 1.10 | 0.11 | (0.89, 1.31) |
| Stop drinking | 1.27 | 0.09 | (1.10, 1.44) |
| Decrease drinking | 0.98 | 0.06 | (0.87, 1.10) |
| **Women** | | |  |
| **WC** | Mean (cm) | ± SE (cm) | 95% CI |
| Stable non-drinking | 3.78 | 0.14 | (3.51, 4.05) |
| Start drinking | 2.66 | 0.25 | (2.16, 3.15) |
| Increase drinking | 3.60 | 0.21 | (3.20, 4.01) |
| Stable drinking | 3.50 | 0.33 | (2.85, 4.15) |
| Stop drinking | 3.21 | 0.24 | (2.74, 3.68) |
| Decrease drinking | 3.23 | 0.23 | (2.79, 3.67) |
| **BMI** | Mean (kg/m^2^) | ± SE (kg/m^2^) | 95% CI |
| Stable non-drinking | 1.49 | 0.06 | (1.38, 1.61) |
| Start drinking | 1.01 | 0.11 | (0.80, 1.23) |
| Increase drinking | 1.29 | 0.09 | (1.11, 1.46) |
| Stable drinking | 1.38 | 0.14 | (1.1,0 1.67) |
| Stop drinking | 1.40 | 0.10 | (1.19, 1.60) |
| Decrease drinking | 1.16 | 0.10 | (0.97, 1.35) |
