# Supplementary material for: Associations of 5-year changes in alcoholic beverage intake with 5-year changes in waist circumference and BMI in the Coronary Artery Risk Development in Young Adults (CARDIA) study
Source: PLoS One. 2023 Mar 8;18(3):e0281722. doi: 10.1371/journal.pone.0281722 (PMC9994756; doi:10.1371/journal.pone.0281722)
Supplement: S3 Table — Values are β coefficients and 95% confidence intervals (95% CI) obtained from longitudinal random effects linear regression models. a Data from men (N = 1,974) and women (N = 2,381) with observations censored for participants with diabetes, hypertension or self-reported cancer during follow-up at the year in which the disease was reported. b Data from men (N = 1,984) and women (N = 2,393) without censoring observations on diabetes, hypertension or self-reported cancer. c Model adjusted for baseline age cohort membership, baseline WC, race and study center and time-varying income, education, smoking status and time-varying changes in marital status, physical activity, and diet quality score. When 5-yr change in BMI was the outcome, models were adjusted for baseline BMI instead of baseline WC. d Model adjusted for baseline age cohort membership, baseline WC, race and study center and time-varying income, education, smoking status and time-varying changes in marital status, physical activity, diet quality and intake of each other alcoholic beverage type. When 5-yr change in BMI was the outcome, models were adjusted for baseline BMI instead of baseline WC. (DOCX) [file pone.0281722.s008.docx]

| **Men** | | | | |
| --- | --- | --- | --- | --- |
|  | Primary Analytic Dataset ^a^ | | Secondary Analytic Dataset ^b^ | |
|  | Model 1^c^ | | Model 1^c^ | |
|  | **5-yr Changes in WC (cm)** | | **5-yr Changes in WC (cm)** | |
| **5-yr Changes in Total Alcoholic Beverage Intake (drinks/wk)** | β | 95% CI | β | 95% CI |
| Stable non-drinking | Ref | - | Ref | - |
| Start drinking | 0.15 | (-0.47 , 0.76) | 0.12 | (-0.45 , 0.69) |
| Increase drinking | 0.10 | (-0.37 , 0.58) | 0.08 | (-0.34 , 0.49) |
| Stable drinking | -0.15 | (-0.84 , 0.54) | 0.03 | (-0.61 , 0.67) |
| Stop drinking | 0.11 | (-0.47 , 0.69) | 0.34 | (-0.2 , 0.88) |
| Decrease drinking | **-0.62** | **(-1.09 , -0.14)** | **-0.52** | **(-0.94 , -0.1)** |
|  | Model 2^c^ | | Model 2^c^ | |
|  | **5-yr Changes in BMI (kg/m^2^)** | | **5-yr Changes in BMI (kg/m^2^)** | |
| **5-yr Changes in Total Alcoholic Beverage Intake (drinks/wk)** | β | 95% CI | β | 95% CI |
| Stable non-drinking | Ref | - | Ref | - |
| Start drinking | -0.09 | (-0.31 , 0.12) | -0.11 | (-0.38 , 0.17) |
| Increase drinking | -0.04 | (-0.2 , 0.13) | -0.05 | (-0.25 , 0.16) |
| Stable drinking | -0.08 | (-0.32 , 0.16) | 0.00 | (-0.31 , 0.32) |
| Stop drinking | 0.09 | (-0.11 , 0.29) | 0.17 | (-0.09 , 0.43) |
| Decrease drinking | **-0.19** | **(-0.36 , -0.03)** | -0.14 | (-0.35 , 0.07) |
|  | Model 1^c^ | | Model 1^c^ | |
|  | **5-yr Changes in WC (cm)** | | **5-yr Changes in WC (cm)** | |
| **5-yr Changes in Drinking Level** | β | 95% CI | β | 95% CI |
| Stable non-drinking | Ref | - | Ref | - |
| Start light/moderate drinking | 0.04 | (-0.49 , 0.57) | 0.12 | (-0.36 , 0.61) |
| Stable light/moderate drinking | -0.24 | (-0.7 , 0.22) | -0.22 | (-0.63 , 0.18) |
| Start excessive drinking | 0.39 | (-0.35 , 1.12) | 0.07 | (-0.6 , 0.73) |
| Stop excessive drinking | **-0.77** | **(-1.51 , -0.03)** | -0.66 | (-1.33 , 0.02) |
| Stop light/moderate drinking | -0.04 | (-0.57 , 0.48) | 0.14 | (-0.34 , 0.62) |
| Stable excessive drinking | -0.11 | (-0.86 , 0.64) | 0.06 | (-0.6 , 0.71) |
|  | Model 2^c^ | | Model 2^c^ | |
|  | **5-yr Changes in BMI (kg/m^2^)** | | **5-yr Changes in BMI (kg/m^2^)** | |
|  | β | 95% CI | β | 95% CI |
| Stable non-drinking | Ref | - | Ref | - |
| Start light/moderate drinking | -0.10 | (-0.29 , 0.08) | -0.09 | (-0.33 , 0.15) |
| Stable light/moderate drinking | -0.10 | (-0.26 , 0.06) | -0.06 | (-0.26 , 0.14) |
| Start excessive drinking | 0.11 | (-0.15 , 0.37) | -0.03 | (-0.35 , 0.3) |
| Stop excessive drinking | -0.11 | (-0.37 , 0.15) | -0.10 | (-0.43 , 0.23) |
| Stop light/moderate drinking | 0.00 | (-0.18 , 0.18) | 0.06 | (-0.17 , 0.3) |
| Stable excessive drinking | -0.22 | (-0.48 , 0.04) | -0.13 | (-0.45 , 0.19) |
|  | Model 3^d^ | | Model 3^d^ | |
| **5-yr Changes in Alcoholic Beverage Intake by Type** | **5-yr Changes in WC (cm)** | | **5-yr Changes in WC (cm)** | |
| **5-yr Changes in Beer** | β | 95% CI | β | 95% CI |
| Stable non-drinking | Ref | - | Ref | - |
| No change in beer drinking | -0.03 | (-0.59 , 0.53) | 0.02 | (-0.49 , 0.52) |
| Increase beer drinking | 0.12 | (-0.4 , 0.64) | 0.26 | (-0.22 , 0.73) |
| Decrease beer drinking | -0.17 | (-0.69 , 0.35) | -0.06 | (-0.53 , 0.42) |
|  | Model 4^d^ | | Model 4^d^ | |
|  | **5-yr Changes in BMI (kg/m^2^)** | | **5-yr Changes in BMI (kg/m^2^)** | |
| **5-yr Changes in Beer** | β | 95% CI | β | 95% CI |
| Stable non-drinking | Ref | - | Ref | - |
| No change in beer drinking | -0.07 | (-0.26 , 0.13) | -0.04 | (-0.29 , 0.21) |
| Increase beer drinking | -0.04 | (-0.22 , 0.15) | 0.02 | (-0.21 , 0.25) |
| Decrease beer drinking | -0.07 | (-0.25 , 0.12) | -0.01 | (-0.24 , 0.22) |
|  | Model 5^d^ | | Model 5^d^ | |
|  | **5-yr Changes in WC (cm)** | | **5-yr Changes in WC (cm)** | |
| **5-yr Changes in Wine** | β | 95% CI | β | 95% CI |
| Stable non-drinking | Ref | - | Ref | - |
| No change in wine drinking | 0.02 | (-0.47 , 0.51) | 0.11 | (-0.33 , 0.55) |
| Increase wine drinking | 0.05 | (-0.52 , 0.62) | 0.14 | (-0.38 , 0.65) |
| Decrease wine drinking | -0.33 | (-0.92 , 0.27) | -0.16 | (-0.7 , 0.39) |
|  | Model 6^d^ | | Model 6^d^ | |
|  | **5-yr Changes in BMI (kg/m^2^)** | | **5-yr Changes in BMI (kg/m^2^)** | |
| **5-yr Changes in Wine** | β | 95% CI | β | 95% CI |
| Stable non-drinking | Ref | - | Ref | - |
| No change in wine drinking | -0.04 | (-0.21 , 0.13) | 0.01 | (-0.21 , 0.22) |
| Increase wine drinking | -0.07 | (-0.27 , 0.12) | -0.04 | (-0.29 , 0.21) |
| Decrease wine drinking | -0.10 | (-0.3 , 0.11) | -0.02 | (-0.28 , 0.25) |
|  | Model 7^d^ | | Model 7^d^ | |
|  | **5-yr Changes in WC (cm)** | | **5-yr Changes in WC (cm)** | |
| **5-yr Changes in Liquor/Mixed Drinks** | β | 95% CI | β | 95% CI |
| Stable non-drinking | Ref | - | Ref | - |
| No change in liquor drinking | -0.23 | (-0.65 , 0.2) | -0.07 | (-0.45 , 0.31) |
| Increase liquor drinking | 0.28 | (-0.23 , 0.8) | 0.15 | (-0.31 , 0.6) |
| Decrease liquor drinking | -0.25 | (-0.76 , 0.25) | -0.26 | (-0.71 , 0.19) |
|  | Model 8^d^ | | Model 8^d^ | |
|  | **5-yr Changes in BMI (kg/m^2^)** | | **5-yr Changes in BMI (kg/m^2^)** | |
| **5-yr Changes in Liquor/Mixed Drinks** | β | 95% CI | β | 95% CI |
| Stable non-drinking | Ref | - | Ref | - |
| No change in liquor drinking | -0.09 | (-0.24 , 0.05) | -0.03 | (-0.21 , 0.16) |
| Increase liquor drinking | 0.00 | (-0.17 , 0.18) | -0.04 | (-0.26 , 0.18) |
| Decrease liquor drinking | -0.09 | (-0.27 , 0.08) | -0.08 | (-0.3 , 0.14) |
| **Women** | | | | |
|  | Primary Analytic Dataset ^a^ | | Secondary Analytic Dataset ^b^ | |
|  | Model 1^c^ | | Model 1^c^ | |
|  | **5-yr Changes in WC (cm)** | | **5-yr Changes in WC (cm)** | |
| **5-yr Changes in Total Alcoholic Beverage Intake (drinks/wk)** | β | 95% CI | β | 95% CI |
| Stable non-drinking | Ref | - | Ref | - |
| Start drinking | **-1.12** | **(-1.69 , -0.56)** | **-1.14** | **(-1.65 , -0.63)** |
| Increase drinking | -0.17 | (-0.67 , 0.32) | -0.15 | (-0.6 , 0.3) |
| Stable drinking | -0.28 | (-0.99 , 0.43) | -0.29 | (-0.93 , 0.35) |
| Stop drinking | **-0.57** | **(-1.11 , -0.03)** | **-0.55** | **(-1.04 , -0.05)** |
| Decrease drinking | **-0.55** | **(-1.08 , -0.02)** | -0.31 | (-0.79 , 0.16) |
|  | Model 2^c^ | | Model 2^c^ | |
|  | **5-yr Changes in BMI (kg/m^2^)** | | **5-yr Changes in BMI (kg/m^2^)** | |
| **5-yr Changes in Total Alcoholic Beverage Intake (drinks/wk)** | β | 95% CI | β | 95% CI |
| Stable non-drinking | Ref | - | Ref | - |
| Start drinking | **-0.48** | **(-0.73 , -0.23)** | **-0.48** | **(-0.71 , -0.26)** |
| Increase drinking | -0.21 | (-0.42 , 0.01) | **-0.21** | **(-0.4 , -0.01)** |
| Stable drinking | -0.11 | (-0.42 , 0.2) | -0.15 | (-0.44 , 0.13) |
| Stop drinking | -0.09 | (-0.33 , 0.14) | -0.14 | (-0.36 , 0.08) |
| Decrease drinking | **-0.33** | **(-0.56 , -0.1)** | -0.21 | (-0.42 , 0) |
|  | Model 1^c^ | | Model 1^c^ | |
|  | **5-yr Changes in WC (cm)** | | **5-yr Changes in WC (cm)** | |
| **5-yr Changes in Drinking Level** | β | 95% CI | β | 95% CI |
| Stable non-drinking | Ref | - | Ref | - |
| Start light/moderate drinking | **-0.78** | **(-1.29 , -0.26)** | **-0.80** | **(-1.26 , -0.34)** |
| Stable light/moderate drinking | **-0.55** | **(-1.05 , -0.04)** | **-0.48** | **(-0.93 , -0.02)** |
| Start excessive drinking | 0.01 | (-0.75 , 0.77) | -0.20 | (-0.89 , 0.5) |
| Stop excessive drinking | -0.77 | (-1.59 , 0.05) | -0.64 | (-1.39 , 0.11) |
| Stop light/moderate drinking | -0.50 | (-1.01 , 0.01) | -0.39 | (-0.85 , 0.07) |
| Stable excessive drinking | -0.31 | (-1.07 , 0.44) | -0.01 | (-0.68 , 0.66) |
|  | Model 2^c^ | | Model 2^c^ | |
|  | **5-yr Changes in BMI (kg/m^2^)** | | **5-yr Changes in BMI (kg/m^2^)** | |
| **5-yr Changes in Drinking Level** | β | 95% CI | β | 95% CI |
| Stable non-drinking | Ref | - | Ref | - |
| Start light/moderate drinking | **-0.42** | **(-0.64 , -0.2)** | **-0.42** | **(-0.63 , -0.22)** |
| Stable light/moderate drinking | -0.20 | (-0.42 , 0.02) | -0.20 | (-0.4 , 0.00) |
| Start excessive drinking | -0.10 | (-0.43 , 0.23) | -0.20 | (-0.5 , 0.11) |
| Stop excessive drinking | -0.27 | (-0.62 , 0.09) | -0.22 | (-0.56 , 0.11) |
| Stop light/moderate drinking | -0.16 | (-0.38 , 0.06) | -0.15 | (-0.35 , 0.06) |
| Stable excessive drinking | -0.33 | (-0.65 , 0) | -0.18 | (-0.48 , 0.12) |
|  | Model 3^d^ | | Model 3^d^ | |
| **5-yr Changes in Alcoholic Beverage Intake by Type** | **5-yr Changes in WC (cm)** | | **5-yr Changes in WC (cm)** | |
| **5-yr Changes in Beer** | β | 95% CI | β | 95% CI |
| Stable non-drinking | Ref | - | Ref | - |
| No change in beer drinking | -0.39 | (-0.89 , 0.11) | -0.39 | (-0.84 , 0.06) |
| Increase beer drinking | -0.41 | (-1 , 0.18) | -0.52 | (-1.06 , 0.01) |
| Decrease beer drinking | -0.50 | (-1.08 , 0.07) | -0.48 | (-1.01 , 0.04) |
|  | Model 4^d^ | | Model 4^d^ | |
|  | **5-yr Changes in BMI (kg/m^2^)** | | **5-yr Changes in BMI (kg/m^2^)** | |
| **5-yr Changes in Beer** | β | 95% CI | β | 95% CI |
| Stable non-drinking | Ref | - | Ref | - |
| No change in beer drinking | -0.15 | (-0.36 , 0.07) | -0.19 | (-0.39 , 0.01) |
| Increase beer drinking | **-0.32** | **(-0.58 , -0.06)** | **-0.36** | **(-0.6 , -0.12)** |
| Decrease beer drinking | -0.20 | (-0.45 , 0.05) | -0.17 | (-0.4 , 0.06) |
|  | Model 5^d^ | | Model 5^d^ | |
|  | **5-yr Changes in WC (cm)** | | **5-yr Changes in WC (cm)** | |
| **5-yr Changes in Wine** | β | 95% CI | β | 95% CI |
| Stable non-drinking | Ref | - | Ref | - |
| No change in wine drinking | **-0.57** | **(-1.1 , -0.04)** | **-0.56** | **(-1.04 , -0.08)** |
| Increase wine drinking | -0.51 | (-1.05 , 0.04) | **-0.56** | **(-1.05 , -0.07)** |
| Decrease wine drinking | -0.16 | (-0.72 , 0.4) | -0.19 | (-0.7 , 0.33) |
|  | Model 6^d^ | | Model 6^d^ | |
|  | **5-yr Changes in BMI (kg/m^2^)** | | **5-yr Changes in BMI (kg/m^2^)** | |
| **5-yr Changes in Wine** | β | 95% CI | β | 95% CI |
| Stable non-drinking | Ref | - | Ref | - |
| No change in wine drinking | **-0.27** | **(-0.5 , -0.04)** | **-0.27** | **(-0.48 , -0.06)** |
| Increase wine drinking | **-0.27** | **(-0.51 , -0.03)** | **-0.30** | **(-0.52 , -0.08)** |
| Decrease wine drinking | -0.04 | (-0.29 , 0.2) | -0.10 | (-0.33 , 0.13) |
|  | Model 7^d^ | | Model 7^d^ | |
|  | **5-yr Changes in WC (cm)** | | **5-yr Changes in WC (cm)** | |
| **5-yr Changes in Liquor/Mixed Drinks** | β | 95% CI | β | 95% CI |
| Stable non-drinking | Ref | - | Ref | - |
| No change in liquor drinking | **-0.54** | **(-0.94 , -0.14)** | **-0.55** | **(-0.9 , -0.19)** |
| Increase liquor drinking | -0.16 | (-0.71 , 0.4) | -0.06 | (-0.55 , 0.44) |
| Decrease liquor drinking | **-0.88** | **(-1.43 , -0.34)** | **-0.69** | **(-1.19 , -0.2)** |
|  | Model 8^d^ | | Model 8^d^ | |
|  | **5-yr Changes in BMI (kg/m^2^)** | | **5-yr Changes in BMI (kg/m^2^)** | |
| **5-yr Changes in Liquor/Mixed Drinks** | β | 95% CI | β | 95% CI |
| Stable non-drinking | Ref | Ref | - | - |
| No change in liquor drinking | **-0.23** | **(-0.4 , -0.06)** | **-0.26** | **(-0.42 , -0.1)** |
| Increase liquor drinking | -0.23 | (-0.48 , 0.01) | -0.17 | (-0.39 , 0.05) |
| Decrease liquor drinking | **-0.33** | **(-0.56 , -0.09)** | **-0.26** | **(-0.48 , -0.04)** |
