# Supplementary material for: Associations of 5-year changes in alcoholic beverage intake with 5-year changes in waist circumference and BMI in the Coronary Artery Risk Development in Young Adults (CARDIA) study
Source: PLoS One. 2023 Mar 8;18(3):e0281722. doi: 10.1371/journal.pone.0281722 (PMC9994756; doi:10.1371/journal.pone.0281722)
Supplement: S1 File — A secondary analytic dataset, that did not include censoring on diabetes, hypertension or self-reported cancer, was created. This secondary dataset included six CARDIA exams (1985–1986, 1990–1991, 1995–1996, 2000–2001, 2005–2006, 2010–2011). All adults with socio-demographic data at baseline were considered eligible participants excluding one participant who withdrew from the study (N = 5,114). As has been done in previous studies, to minimize bias resulting from illness that may affect body weight, we excluded participants with hypertension (SBP/DBP ≥ 140/≥90 mm Hg or taking medication for elevated BP; N = 145) diabetes (fasting glucose ≥126 mg/dL or taking medication for diabetes; N = 29) or cancer (self-reported diagnosis; N = 137) at baseline. (29,37) Further, we excluded participants who were missing data on diabetes, hypertension, self-reported cancer diagnoses (N = 9) at each exam and those missing waist circumference (N = 17) or BMI (N = 4) at exam year 0. We also excluded participants who attended only one of the six exams used in the current study exam (N = 351). Individuals with excluded observations at every exam were excluded from the analytic sample (N = 45). For individuals included in the analytic sample, observations were excluded at given exam years if participants were pregnant or breastfeeding (observations = 305) or had implausible energy intakes (<600 kcal/d or >6000/d kcal for women and <800 kcal/d or >8000 kcal/d for men) (observations = 393) at an exam or if they were missing exposure (observations = 1347 alcohol intake), outcome (observations = 34 WC, 131 BMI), or covariate data at a given exam year (observations = 1 education, 6 marital status, 102 smoking, 50 physical activity, 1,159 dietary intake). Our final analytic sample consisted of 4,377 participants (men and women) n = 4,241 at year 0; 3,771 at year 5; 2,966 at year 10; 2,717 at year 15; 2,513 at year 20 and 2,463 at year 25 for a total 18,671 person observations [8]. (DOCX) [file pone.0281722.s009.docx]

**Supplemental Methods and Analyses**

A secondary analytic dataset, that did not include censoring on diabetes, hypertension or self-reported cancer, was created. This secondary dataset included six CARDIA exams (1985–1986, 1990-1991, 1995-1996, 2000-2001, 2005-2006, 2010-2011). All adults with socio-demographic data at baseline were considered eligible participants excluding one participant who withdrew from the study (N=5,114). As has been done in previous studies, to minimize bias resulting from illness that may affect body weight, we excluded participants with hypertension (SBP/DBP ≥ 140/≥90 mm Hg or taking medication for elevated BP; N=145) diabetes (fasting glucose ≥126 mg/dL or taking medication for diabetes; N=29) or cancer (self-reported diagnosis; N=137) at baseline. (29,37) Further, we excluded participants who were missing data on diabetes, hypertension, self-reported cancer diagnoses (N=9) at each exam and those missing waist circumference (N=17) or BMI (N=4) at exam year 0. We also excluded participants who attended only one of the six exams used in the current study exam (N=351). Individuals with excluded observations at every exam were excluded from the analytic sample (N=45). For individuals included in the analytic sample, observations were excluded at given exam years if participants were pregnant or breastfeeding (observations=305) or had implausible energy intakes (<600 kcal/d or >6000/d kcal for women and <800 kcal/d or >8000 kcal/d for men) (observations= 393) at an exam or if they were missing exposure (observations=1347 alcohol intake), outcome (observations = 34 WC, 131 BMI) , or covariate data at a given exam year (observations =1 education, 6 marital status, 102 smoking, 50 physical activity, 1,159 dietary intake). Our final analytic sample consisted of 4,377 participants (men and women) n=4,241 at year 0; 3,771 at year 5; 2,966 at year 10; 2,717 at year 15; 2,513 at year 20 and 2,463 at year 25 for a total 18,671 person observations. [8]
